# Glycolysis Is Governed by Growth Regime and Simple Enzyme Regulation in Adherent MDCK Cells

**DOI:** 10.1371/journal.pcbi.1003885

**Published:** 2014-10-16

**Authors:** Markus Rehberg, Joachim B. Ritter, Udo Reichl

**Affiliations:** 1 Max Planck Institute for Dynamics of Complex Technical Systems, Magdeburg, Germany; 2 Otto von Guericke University Magdeburg, Chair of Bioprocess Engineering, Magdeburg, Germany; ETH Zurich, Switzerland

## Abstract

Due to its vital importance in the supply of cellular pathways with energy and precursors, glycolysis has been studied for several decades regarding its capacity and regulation. For a systems-level understanding of the Madin-Darby canine kidney (MDCK) cell metabolism, we couple a segregated cell growth model published earlier with a structured model of glycolysis, which is based on relatively simple kinetics for enzymatic reactions of glycolysis, to explain the pathway dynamics under various cultivation conditions. The structured model takes into account *in vitro* enzyme activities, and links glycolysis with pentose phosphate pathway and glycogenesis. Using a single parameterization, metabolite pool dynamics during cell cultivation, glucose limitation and glucose pulse experiments can be consistently reproduced by considering the cultivation history of the cells. Growth phase-dependent glucose uptake together with cell-specific volume changes generate high intracellular metabolite pools and flux rates to satisfy the cellular demand during growth. Under glucose limitation, the coordinated control of glycolytic enzymes re-adjusts the glycolytic flux to prevent the depletion of glycolytic intermediates. Finally, the model's predictive power supports the design of more efficient bioprocesses.

## Author Summary

Glycolysis generates biomass precursors and energy from sugars and is therefore a key element in the metabolism of mammalian cells. Changes in its activity greatly affect cellular function which is often recognized as metabolic disease but also as opportunity for the design of efficient bioprocesses. Metabolic research discovered that continuously growing mammalian cells often exhibit a high glycolytic activity but also delivered seemingly endless facets in the pathway operation. The latter call for a systems-level understanding regarding capacity and regulation for a broad range of cultivation conditions. In this work, we couple a cell growth model to a simple kinetic description of glycolysis to consistently explain intracellular metabolite pool dynamics of the Madin-Darby canine kidney cell line over a variety of experiments and time scales while considering the growth status and cultivation history of the cells. We argue that the many different dynamics in glycolysis result from an interplay between a growth-dependent sugar uptake together with simple intrinsic enzyme regulation.

## Introduction

The primary metabolism of cells is essential for cell growth and maintenance. Glycolysis is a central element of the primary metabolic activity and supplies anabolic pathways with precursors and cellular energy in form of ATP. The detailed *in vitro* characterization of glycolytic enzymes, such as hexokinase (HK), phosphofructokinase (PFK) and pyruvate kinase (PK), with respect to their catalytic properties in the presence of substrates, products and allosteric effectors represents an initial step towards a kinetic description of metabolic phenomena of cells [1]–[3]. Dynamic mathematical models of glycolysis have been developed for many different organisms such as *Escherichia coli*, yeast, or mammalian cells. Such models range from simple to full kinetic descriptions with the intention to study specific observations, e.g., metabolic steady states [4]–[6] perturbation of substrates [7]–[9] or enzymes [10], flux sensors [11], oscillations in glycolysis [12], the glucose uptake system [13], or the link of liver cell glycolysis with blood glucose homeostasis [14], [15]. Although in many cases the existing experimental data sets do not allow for a full validation of highly complex models in a broad physiological context, there is a clear benefit regarding the integration of complex regulatory mechanisms, which helps to explain general phenomenological aspects that are typically found in the respective organism. However, an apparently complex metabolic behavior must not result from complex regulatory mechanisms [16]. In case of glycolysis, it seems that few regulatory mechanisms dominate the dynamics of intracellular metabolite pools and readily explain salient features of experimental observations [17]. Furthermore, with an increasing number of powerful assays, e.g. to determine intracellular metabolite concentrations or to measure enzyme activities in yeast and animal cells (e.g. [18]–[21]), changes in glycolytic activity for cell growth or substrate perturbations can be monitored at an unprecedented level. Based on the additional quantification of extracellular metabolite changes and cell number measurements a systematic analysis of basic dynamics of glycolysis for various cultivation conditions is possible.

Recently, we reported that adherent MDCK cells cultivated in two different media not only show similar and reproducible dynamics of many intracellular metabolite pools but also that changes in their concentrations are growth phase-dependent [22]. With the aim to elucidate the interplay between enzyme and growth regime-mediated regulation of glycolysis, a segregated cell growth model has been developed, which captures experimental observations during cell growth phases regarding number increase, diameter change and uptake of substrates [23].

Here, we couple this segregated cell growth model to a structured model which incorporates a simple kinetic description of glycolysis and focusses on a few well-known enzymatic properties to elucidate the control of glycolysis. In addition, the linkage to the pentose phosphate pathway and the glycogenesis are taken into account. We evaluate the model's ability to reflect changes in intracellular metabolite pools for a variety of cultivation conditions using a single set of parameters. This includes the transition from the exponential to the stationary cell growth phase, the fast replacement of medium by PBS at different time points of cultivation, and a substrate pulse experiment. Afterwards we discuss the influence of growth regime, changes in extracellular metabolite concentrations, and activity of key enzymes on the control of glycolysis. In addition, aspects of hierarchical regulation are addressed which, taken together, improve our understanding of the metabolism of fast proliferating cells. Finally, options for the modulation of metabolic activity are evaluated regarding the design and optimization of cell culture processes as well as the study of metabolic diseases.

## Results

### Glycolytic activity under changing growth regimes

**Figure 1:**
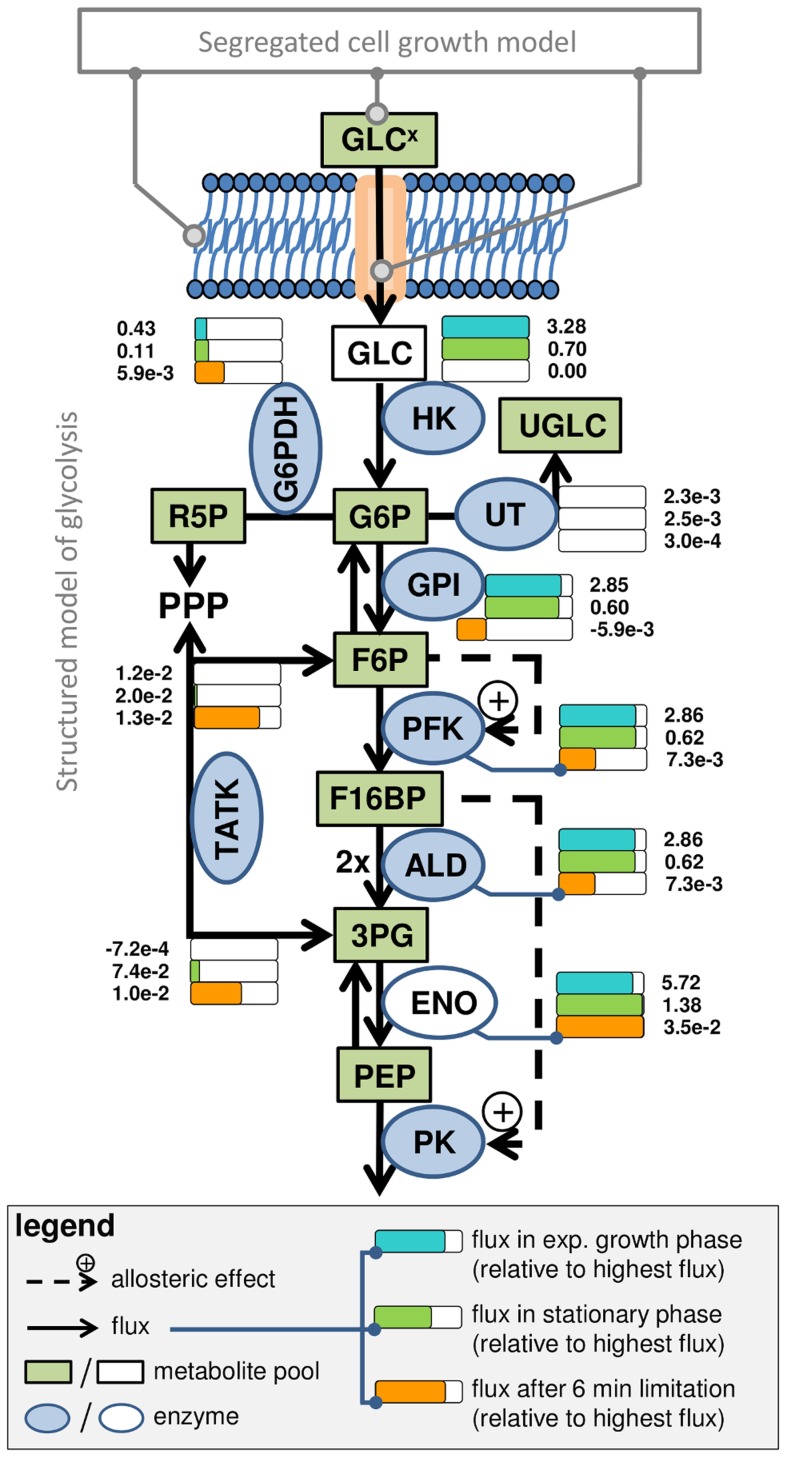
Scheme of glycolysis model (B) and its link to the segregated cell growth model (A) established previously [23]. Green boxes represent metabolite pools that were quantified experimentally while white ones were not measured. Enzymes are shown as ellipses with blue background if the maximum enzyme activity was measured *in vitro* and with white background otherwise. Reactions and their directions are shown as arrows. Dashed arrows represent allosteric regulation of enzymes by metabolites. The activity of the enzymes relative to the highest activity in glycolysis (see legend) is attached to the corresponding reactions/enzymes and expressed by colored bars (blue: cell growth at 24 h of Cult1; green: cell maintenance at 100 h of Cult1; orange: limitation at 6 min). Absolute flux rates (unit: mmol L^−1^ min^−1^) are given next to the bars. GLC^x^ extracellular glucose; GLC glucose; G6P glucose 6-phosphate; UGLC uridyl diphosphate glucose; R5P ribose 5-phosphate; PPP pentose phosphate pathway; F6P fructose 6-phosphate; F16BP fructose 1,6-bisphosphate; 3PG 3-phosphoglyceric acid; PEP phosphoenol pyruvate; HK hexokinase; UT UTP-glucose-1-phosphate uridylyltransferase; G6PDH glucose 6-phosphate dehydrogenase; GPI glucose phosphate isomerase; ALD aldolase; ENO enolase; PK pyruvate kinase.

In three independent experiments, adherent MDCK cells were grown in 6-well plates with the serum-containing medium GMEM-Z, which provides sufficient amounts of extracellular substrates over the chosen cultivation time. Therefore, cell growth occurs with maximum rate until the available surface becomes limiting [23]. The experimental data of intracellular metabolite pools is taken from Rehberg et al. [22], and analyzed in the following using the model described in the Materials & Methods section (see section “Model and simulation”). The model focuses on intermediates that were measured experimentally and is composed of a concise set of enzyme kinetics with few regulatory mechanisms. A schematic overview of the considered enzyme reactions, the measured metabolite pools and maximum *in vitro* enzyme activities, and the coupling to the previously developed segregated model of cell growth [23] is given in Fig. 1.

#### Upper glycolysis

**Figure 2:**
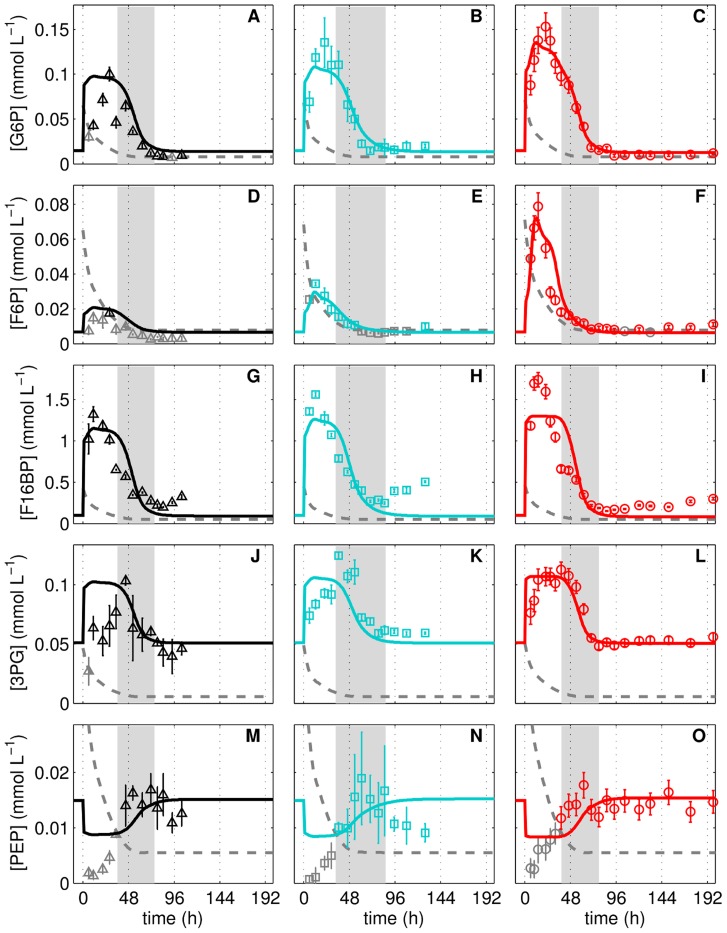
Metabolites pools of glycolysis during adherent MDCK cell cultivation. Glucose 6-phosphate (**A**–**C**), fructose 6-phosphate (**D**–**F**), fructose 1,6-bisphosphate (**G**–**I**), 3-phosphoglyceric acid (**J**–**L**) and phosphoenolpyruvate (**M**–**O**) concentrations in three independent MDCK cell cultivations (Cult1 ▵, Cult2 □, Cult3 ○) in 6-well plates and GMEM-Z. Data and error bars represent mean and standard deviation of three wells. Dashed lines are the limit of quantification (LOQ; data below LOQ marked in grey). Lines represent the respective simulation result based on the experiment-specific parameters in Table 1 and parameters in Table 2. The intermediate growth phase (95%–5% proliferating cells) is indicated as grey bar for the respective cultivation.

The model takes into account that the adherent MDCK cells used for inoculation of cultivation I, II, and III (Cult1 (▵), Cult2 (□) and Cult3 (○)) originate from a preculture that has reached the stationary growth phase (e.g. Cult1—3 with t>86 h). The corresponding metabolic steady state is depicted in the time interval from −20 h to 0 h (Fig. 2), and the values of the model simulation are shown in Table 1. In particular, we assume that this metabolic status is reproducibly achieved in the preculture (which is the case in all three cultivations) and represents the metabolic starting point for the batch cultivation experiments Cult1–3. Note that variations of ±20% in initial conditions of intracellular metabolite concentrations have no impact on the simulation results, since the activity of glycolysis adjusts the cellular pools within seconds. However, using a simulated initial metabolic status reduces the number of parameters that require optimization (discussed in section “Model coupling and simulation”), avoids an artificial model behavior due to an inconsistent assignment of initial conditions and, most importantly, is biologically more relevant as cells indeed originate from a stationary growth phase with constant metabolite pools (e.g. Cult1 at t = 200 h).

**Table 1:** Initial conditions for the structured model comprising metabolic status, growth status and culture conditions for the simulated experiment.

	Cult1 Preculture^a^	Cult2 Preculture^a^	Cult3 Preculture^a^	Pred. Preculture^a^	Lim1 (Cult1 at t^*^ = 48 h)	Lim2 (Cult1 at t^*^ = 60 h)	Pulse (Cult1 at t^*^ = 30 h)	Unit
Metabolic status								
[GLC]	1.18e-4	1.18e-4	1.18e-4	1.18e-4	7.96e-4	3.30e-4	0.00	mmol L^−1^
[G6P]	1.46e-2	1.46e-2	1.46e-2	1.46e-2	6.03e-2	3.25e-2	1.15e-3	mmol L^−1^
[F6P]	6.66e-3	6.66e-3	6.66e-3	6.66e-3	1.19e-2	8.86e-3	2.31e-3	mmol L^−1^
[R5P]	5.76e-3	5.76e-3	5.76e-3	5.76e-3	2.36e-2	1.28e-2	4.55e-4	mmol L^−1^
[UGLC]	0.25	0.25	0.25	0.25	0.36	0.34	1.97e-1	mmol L^−1^
[F16BP]	1.05e-1	1.05e-1	1.05e-1	1.05e-1	0.63	0.28	1.64e-3	mmol L^−1^
[3PG]	4.74e-2	4.74e-2	4.74e-2	4.74e-2	7.67e-2	5.78e-2	8.86e-2	mmol L^−1^
[PEP]	1.42e-2	1.42e-2	1.42e-2	1.42e-2	1.03e-2	1.22e-2	3.53e-2	mmol L^−1^
Growth status								
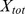	0.40e6	0.41e6	0.48e6	0.30e6^c^	2.10e6	2.69e6	1.11e6	cells
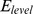	1.08^b^	1.04^b^	0.92^b^	1.00	1.08	1.08	1.08	-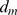	15.68	15.31	14.34	15.48^c^	n/a	n/a	n/a	µm
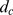	22.93	24.86	20.98	21.12^c^	n/a	n/a	n/a	µm
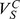	n/a	n/a	n/a	n/a	3.04e-12	2.53e-12	3.46e-12	L/cell
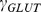	n/a	n/a	n/a	n/a	0.52	0.27	0.77	-
Culture conditions								
[GLC^x^]	31.04	29.25	31.95	2.31^c^	24.22	22.58	30.00	mmol L^−1^
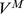	4e-3	4e-3	4e-3	4e-3	3e-7^b^	3e-7^b^	4e-3	L

The estimated cultivation history of cells is given below the experiment name.

a precultures were carried out in T-flasks (Cult1, Cult2, Pred.) or roller bottles (Cult3) which caused slight differences in culture conditions and growth status of cells (see Rehberg et al. [23]). Initial values for intracellular metabolite concentrations (metabolic status) are taken from the steady state value of Cult1 at 200 h determined via model simulations.

b estimated during model fitting.

c see supporting information 4.

**Figure 3:**
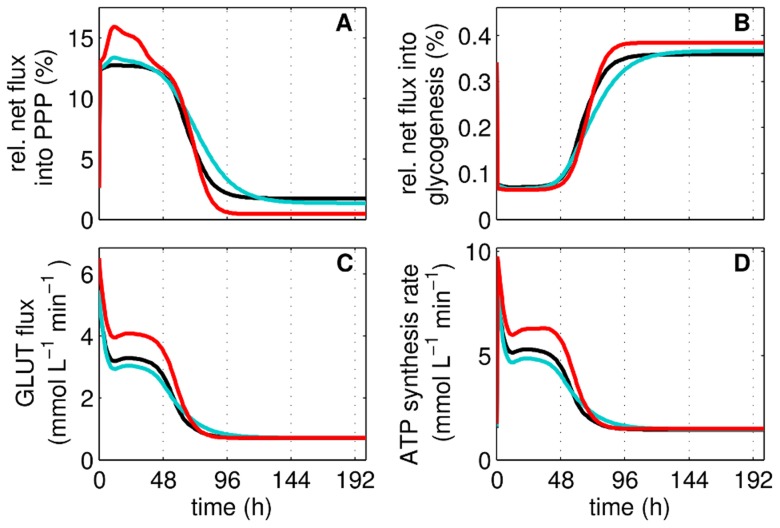
Estimated fluxes for energy and precursors production during adherent MDCK cell cultivation. Net flux into pentose phosphate pathway (PPP) relative to glucose transport flux (**A**, see section “Exchange of glycolytic metabolites through other reactions”), net flux into glycogenesis relative to glucose transport flux (**B**), glucose transport flux (**C**), and ATP production rate (**D**) are simulated for the three cultivations (Cult1 – 3) and shown in the color code of Fig. 2.

With onset of the cell growth phase (t = 0 h, of Fig. 2), the simulation of the three experiments follows the measured peak-like behavior of glucose 6-phosphate (G6P), fructose 6-phosphate (F6P) and fructose 1,6-bisphosphate (F16BP) concentrations (Fig. 2A–I), which together form the upper part of glycolysis. The maximum is reached at around 24 h of cultivation and roughly coincides with the onset of cell growth inhibition (indicated by the grey bar). In the model, the peak results from high cell volume-specific glucose uptake rates and low maximum cell volume-specific enzyme activities. In the intermediate growth phase (34–86 h), the concentrations of all three metabolites drop to their initial level (−20 h to 0 h, Fig. 2A–I). Due to the tight coupling of cell growth to glycolysis, the model considers experiment-specific differences such as the cell number 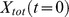 used for inoculation as well as the minimum and maximum mean cell diameter (d_m_ and d_c_; Table 1), which have the strongest effect on time point and height of the peak. In addition, we performed a sensitivity analysis to investigate the influence of parameters and initial conditions on the model behavior (supporting information, Fig. S1). Differences in growth and metabolic status of cells used for inoculation of cultivation experiments indicate that the cells are obviously not identical. It is therefore likely that not only the cell size but also the enzyme level (E_level_) differs to a certain degree. For the quantification of cell number-specific enzyme activities, Janke et al. [19] measured six biological replicates and found a mean relative standard deviation for the activities of about ±8%. Therefore, we introduce the E_level_ as an experiment-specific value to modulate the maximum catalytic activity of every enzyme in the model with a range from 0.92 to 1.08 (Eq. (4)). The model suggests that the cells with the lowest diameters (d_m_, d_c_), i.e. Cult3 (○), also have the lowest E_level_ (Table 1). Besides variations due to assay noise, the experiment-specific differences in cell number, mean cell diameter and E_level_ explain batch-to-batch variations such as the lower peak height for Cult1 (▵, Fig. 2A, D, G, J, M), a medium peak height for Cult2 (□, Fig. 2B, E, H, K, N) and an increased peak height for Cult3 (○, Fig. 3C, F, I, L, O), which is most prominent for F6P. An exemplary intracellular flux from glycolysis into associated pathways is shown for Cult1 in Fig. 1. During cell growth the flux through HK (3.28 mmol L^−1^ min^−1^) is roughly five times higher than during stationary growth (0.7 mmol L^−1^ min^−1^; Fig. 1) while 13% of the generated G6P is transferred into the pentose phosphate pathway (PPP) for synthesis of macromolecules, purines and pyrimidines. The transfer of G6P into glycogenesis via the UTP-glucose-1-phosphate uridylyltransferase (UT) mediated reaction as well as the exchange of F6P with the PPP via the transaldolase and transketolase mediated reaction (TATK) show very low activities (<1% of the flux through HK, Fig. 1).

#### Lower glycolysis

The level of 3-phosphoglyceric acid (3PG) follows the peak-like behavior of upper glycolysis albeit with a two-fold increase only, which is quite similar among the three cultivations (Fig. 2J–L). The data of Cult1 (▵) have a larger standard deviation which complicates the assessment of the peak-like behavior. The data for phosphoenol pyruvate (PEP) are below the limit of quantification until 48 h of cultivation (indicated by grey symbols) but still support the hypothesis of a fast drop at the beginning of cultivation with a slow but steady increase until the stationary growth phase begins (Fig. 2M–O, 50–200 h). Under consideration of these data points, the model similarly suggests a decrease and increase in PEP levels on the basis of an allosteric feed-forward activation of PK by F16BP. Otherwise, a straight line would suffice to describe the data. In the stationary growth phase, the simulation result is slightly above the data points as higher levels of PEP are advantageous for fitting of the perturbation experiments (see section “Response of glycolysis to perturbation experiments). In the model, the lower part of glycolysis shows a four-fold increase in the activity during cell growth (5.7 mmol L^−1^ min^−1^) compared to the stationary growth phase (1.39 mmol L^−1^ min^−1^). Interestingly, during cell maintenance most of the PPP metabolites, synthesized by glucose 6-phosphate dehydrogenase (G6PDH), are fed back into glycolysis through the TATK mediated reactions (Fig. 1). Hence, most of the glucose influx during the stationary growth phase is converted to pyruvate (PYR).

#### Exchange of glycolytic metabolites through other reactions

The products of the PPP are used for nucleotide and nucleic acid synthesis, production of macromolecules and yield NADPH for the synthesis of fatty acids. The net flux into the PPP, is 3% to 15% of the glycolytic flux (G6PDH-TATKF6P-0.5TATK3PG relative to the glucose transporter (GLUT) flux) depending on the cell growth phase (Fig. 3A), and fulfills the constraint to be in the range of 0% to 40% (see supporting information 2). Glycogenesis mainly generates glycogen and the relative net flux, which is branched off from glycolysis for this pathway (UT relative to GLUT), is less than 0.1% during cell growth and increases to 0.4% during cell maintenance (Fig. 3B). The activity of the glucose transporters during cell cultivation has an initial peak followed by a stepwise decrease (Fig. 3C). The first decrease is a product of an immediate start of a high extracellular glucose (GLC^x^) uptake under a slowly increasing cell-specific volume. Therefore, the model suggests a relatively high consumption by the cell at initial times of cultivation. The second decrease results from a reduced cellular demand of GLC^x^ due to growth inhibition. The net production of ATP by glycolysis is calculated by adding the flux through PK and phosphoglycerate kinase (here ENO, see supporting information 3) minus the flux through HK and PFK. In the simulation, the net production rate of ATP is strongly correlated to the GLUT activity (Fig. 3D). Furthermore, glycolysis produces 1.5–10 mmol L^−1^ min^−1^ of ATP depending on the cellular growth status.

### Response of glycolysis to perturbation experiments

#### Limitation experiments

**Figure 4:**
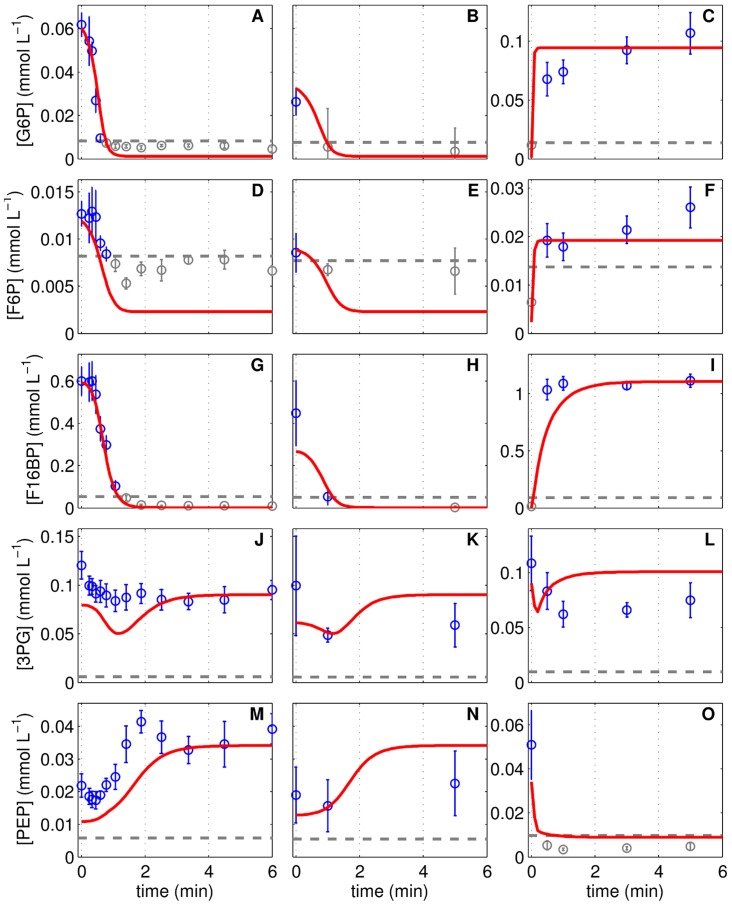
The response of intracellular metabolite pools to perturbation experiments. Glucose 6-phosphate (**A**–**C**), fructose 6-phosphate (**D**–**F**), fructose 1,6-bisphosphate (**G**–**I**), 3-phosphoglyceric acid (**J**–**L**) and phosphoenolpyruvate (**M**–**O**) concentrations of three independent perturbation experiments with MDCK cells in 6-well plates. Cells originating from a cultivation experiment (see Table 1) were deprived of extracellular nutrients by removal of medium and addition of phosphate buffered saline, shown in the first column (Lim1, **A**,**D**,**G**,**J**,**M**) and second column (Lim2, **B**,**E**,**H**,**K**,**N**). After a 2 h limitation, PBS was exchanged by fresh medium (Pulse, **C**,**F**,**I**,**L**,**O**). Data (○) and error bars represent mean and standard deviation of three wells, respectively. Dashed lines are the limit of quantification (LOQ; data below LOQ marked in grey). Lines represent the respective simulation result based on the experiment-specific parameters in Table 1 and parameters in Table 2.

At a certain time point of cultivation the medium was replaced by PBS, which essentially removes all substrates and by-products. Unexpectedly, the intracellular metabolite pools of upper glycolysis, i.e., G6P, F6P and F16BP, show different starting concentrations in the first (Lim1; Fig. 4A,D,G) and the second limitation experiment (Lim2; Fig. 4B,E,H). Obviously the metabolic status of cells is not identical although taken from a similar time point of cultivation. As the metabolic status of cells greatly depends on the growth phase (see section “Glycolytic activity under changing growth regimes”), we assume that that the cells used for the limitation experiment originate from different time points of cultivation (t*, using the Cult1 simulation as the origin of cells). The resulting difference in the initial metabolite pool levels upon selection of a t* (Lim1: 48 h, Lim2: 60 h, see Table 1) allows to resemble the measured initial metabolic status of the perturbation experiments (Fig. 4). Choosing Cult2 or Cult3 as a starting point for simulations yields similar simulation results. Furthermore, the model takes into account that 3×10^−7^ L medium remain on the cellular surface and in the intercellular space as a glycolytic activity of 3.28 mmol L^−1^ min^−1^ would, for example, deplete the G6P pool within a second, which is obviously not the case (Fig. 4A, 4B). However, it takes about only one minute until the corresponding metabolite pools drop below the limit of quantification. Interestingly, F6P and G6P are still detected while the pool of F16BP is fully consumed. According to the model, a flux from PPP to F6P of about 0.013 mmol L^−1^ min^−1^ is sufficient to maintain the F6P and G6P pool under a reversed activity of the glucosephosphate isomerase (GPI; Fig. 1). However, G6PDH transfers G6P back into the PPP and completes a very low cyclic metabolite exchange between both pathways. The activity of PFK is reduced under low F6P levels, but a slight flux remains and generates 3PG (Fig. 1). Overall, we conclude that the model is in good agreement with experimental data for cells under glucose limitation, especially for those above the limit of quantification.

In the lower part of glycolysis, 3PG and PEP remain comparatively constant or even increase in concentration until reaching a steady state after 3 min (Fig. 4J,K,M,N). In the model, the increase in PEP results from a reduction in the PK activity due to decreasing F16BP levels (Fig. 1). The initial concentration of PEP measured in both experiments is higher than in simulations but also higher than the levels found in the cultivation experiment (Fig. 2). To improve the fitting of the Lim1 and Lim2 experiments, the model realized slightly higher final PEP levels in the cultivation experiments than measured experimentally. The simulation of 3PG showed a short drop and a subsequent increase after 1 min of glucose limitation which may also be present in the data although to a lesser extent.

#### Pulse experiments

The pulse experiment follows the limitation experiment, which used cells from approximately 32 h of Cult1, by replacing the PBS after two hours of incubation with fresh medium providing glucose and other substrates. The model suggests that glycolysis almost immediately (it takes 1.4 s to achieve a 5% flux through PK) starts with the conversion of glucose to pyruvate and that the metabolite pools reach a steady state after one to two minutes (Fig. 4C,F,I,L,O). Such a fast increase in glycolytic intermediates was also observed for sarcoma 180 ascites tumor cells [24]. As a result, the dynamics are mirroring the limitation experiment with increasing levels in upper glycolysis (Fig. 4C,F,I) and decreasing PEP pools (Fig. 4O) due to the feed-forward activation of PK by F16BP. However, the slight but continuous increase of G6P and F6P pools is not reflected by the model and also 3PG, which remains more or less constant in the simulation with a small drop at 0.5 min, is slightly different compared to the data (Fig. 4L). However, the model simulation resembles at t = 6 min the metabolic status of Cult1 at 32 h of cultivation, which fits most of the pulse experiment data.

### Link to pentose phosphate pathway and glycogenesis

**Figure 5:**
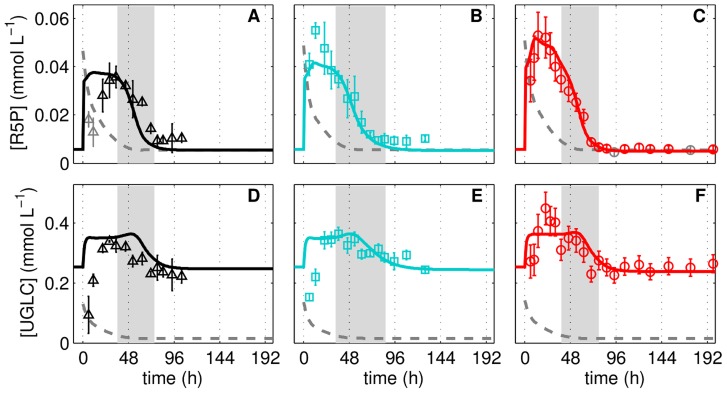
Intracellular metabolite pools of pentose phosphate pathway and glycogenesis during adherent MDCK cell cultivation. Ribose 5-phosphate (**A**–**C**) and uridyl diphosphate glucose (**D**–**F**) concentrations of three independent MDCK cell cultivations (Cult1 ▵, Cult2 □, Cult3 ○) in 6-well plates and GMEM-Z. Data and error bars represent mean and standard deviation of three wells, respectively. Dashed lines are the limit of quantification (LOQ; data below LOQ marked in grey). Lines represent the respective simulation result based on the experiment-specific parameters in Table 1 and parameters in Table 2. The intermediate growth phase (95%–5% proliferating cells) is indicated as grey bar for the respective cultivation.

**Figure 6:**
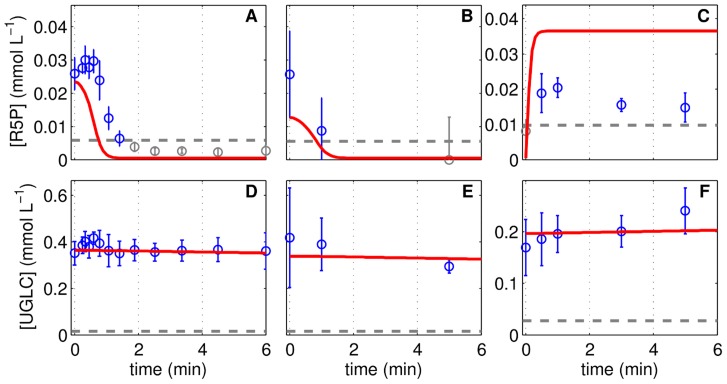
The response of metabolite pools of pentose phosphate pathway and glycogenesis to perturbation experiment. Ribose 5-phosphate (**A**–**C**) and uridyl diphosphate glucose (**D**–**F**) concentrations in three independent perturbation experiments with MDCK cells in 6-well plates. Cells originating from a cultivation experiment (see Table 1) were deprived of extracellular nutrients by removal of medium and addition of phosphate buffered saline, shown in the first column (Lim1, **A**,**D**) and second column (Lim2, **B**,**E**). After 2 h of incubation, PBS was exchanged by fresh medium (Pulse, **C**,**F**). Data (○) and error bars represent mean and standard deviation of three wells, respectively. Dashed lines are the limit of quantification (LOQ; data below LOQ marked in grey). Lines represent the respective simulation result based on experiment-specific parameters in Table 1 and parameters in Table 2.

The implemented G6PDH and UT mediated conversion of G6P are entry points into the PPP and the glycogenesis, respectively. They eventually fuel the pools of ribose 5-phosphate (R5P) and uridyl diphosphate glucose (UGLC) and implementation of simple degradation reactions (Eq. (11), (12)) allows assessing the consistency between the flux through G6PDH and the R5P pool as well as between the flux through UT and the UGLC pool. The expense of an additional model parameter for the ribose 1,5-bisphosphate phosphokinase (RDPK) and glycogen synthase (GLYS), which both represent only one of the possible degradation reactions, enables the model to reflect the dynamics of R5P and UGLC during cell cultivation (Fig. 5). Note that in contrast to other intracellular metabolites, UGLC is diluted by cell volume growth to a visible extent, which reduces the typical peak-like behavior compared to other metabolites (Fig. 5D–F). During the limitation experiment, the pool of R5P decreases later than suggested by the model yet with similar dynamics. During the pulse experiment, the level of R5P is lower than suggested by the model (Fig. 6A,B,C). In both cases, the differences between experimental data and simulation results might be due to network properties of the PPP, which are not considered by the model (for instance, the high number of reversible reactions, and the linkage of its intermediates to the biosynthesis machinery). The data for UGLC shows only a minor decrease and a minor increase during the limitation and pulse experiments, respectively, which is described by the model (Fig. 6D,E,F) and clearly attributed to the low pathway activity (Fig. 1).

### Estimations and predictions for the metabolic activity

#### ATP and biomass precursors generation under modulation of the glucose transport activity

**Figure 7:**
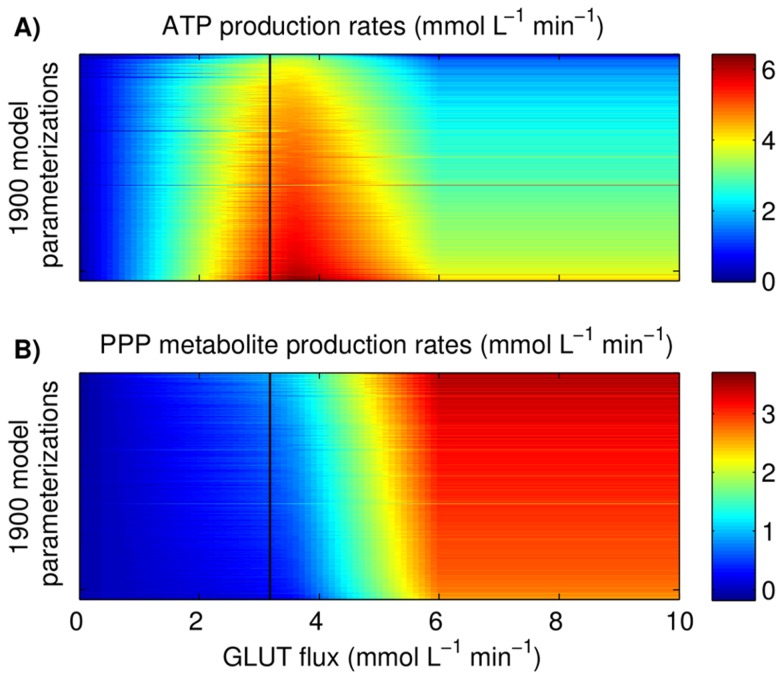
Impact of *in silico* GLUT modulation on (A) ATP and (B) pentose phosphate pathway (PPP) production rates. At total of 1900 model parameterizations (0.025–0.975 quantile of 2000 model parameterizations) were assessed for the GLUT modulation and were derived from the optimal result of each bootstrap run, which was also the basis for estimation of the parameter confidence intervals of Table 2. The colored bars on the right hand show the respective production rate; the black vertical line represents the original GLUT activity of cells of Cult1 at 24 h.

The correlation of the (GLUT) activity and the ATP synthesis rate in Fig. 3C and Fig. 3D already indicates that the glycolytic flux is controlled by the GLUT during cell cultivation. Modulation of the GLUT is not only a target for the improvement of production cell lines but also an approach considered for cancer treatment with the intention to interfere with the high metabolic activity of cells, and eventually with tumor growth. For the subsequent analysis of glycolysis by *in silico* modulation of the GLUT activity we chose cells from Cult1 at 24 h of cultivation. As before, the net production rate of ATP is estimated as the sum of the flux through PK and phosphoglycerate kinase (here ENO, see supporting information 3) minus the flux through HK and PFK. The net production of PPP metabolites is the flux through G6PDH minus the flux through TATKF6P and half of TATK3PG as it yields only three carbon sugars. For the analysis, we also consider the impact of the parameter uncertainty by using all model parameterization derived from the bootstrap method (see section “Computation”), which in sum comprise 2000 parameter sets describing our data for glycolysis. The modulation of the GLUT activity in all these model parameterization was chosen to range from 0–10 mmol L^−1^ min^−1^, which exceeds the typical uptake rates determined during cell growth and substrate limitation (e.g. in Cult1: 0–3 mmol L^−1^ min^−1^). The resulting steady state production rates of ATP and PPP metabolites were sorted in increasing ATP production rate and are shown in Fig. 7. As expected, the ATP and PPP metabolite production rate increases with higher fluxes through GLUT up to about 4 mmol L^−1^ min^−1^, depending on the model parameterization. A further increase to 6 mmol L^−1^ min^−1^ saturates the PFK (for cells of Cult1 at 24 h). The resulting shift of the metabolic flux into the PPP further increases the synthesis of metabolites but impairs the glycolytic ATP production. The increase in PPP metabolite production results exclusively from an enhanced flux through G6PDH, which, in cooperation with other enzymes, also yields NADPH. As a result, the production of NAPDH correlates linearly with the PPP metabolite production, which are both essential for biosynthesis. However, for a flux through GLUT>6 mmol L^−1^ min^−1^, the HK becomes saturated as well and a further increase of the GLUT activity results in accumulation of intracellular glucose (GLC).

#### Prediction of metabolic activity during growth in different medium

**Figure 8:**
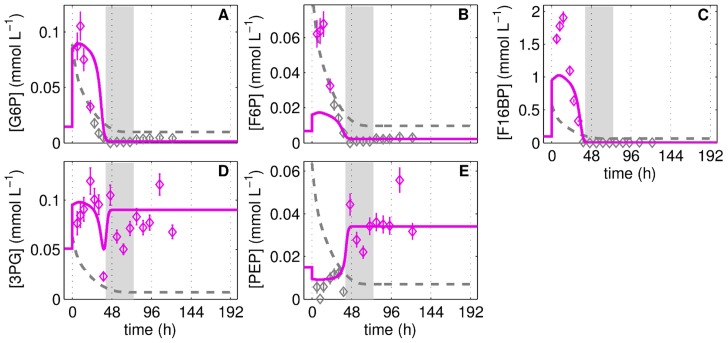
Prediction of glycolytic metabolite pools during cultivation of adherent MDCK cells in DMEM with 2.5 mmol L^−1^ extracellular glucose. Glucose 6-phosphate (**A**), fructose 6-phosphate (**B**), fructose 1,6-bisphosphate (**C**), 3-phosphoglyceric acid (**D**) and phosphoenolpyruvate (**E**) concentrations during MDCK cell cultivations in 6-well plates and DMEM medium with 2.5 mmol L^−1^ extracellular glucose. Data (◊) and error bars represent mean and standard deviation of three wells. Dashed lines are the limit of quantification (LOQ; data below LOQ marked in grey). Lines represent the model prediction based on the modifications of the cell growth model described in the supporting information 4 and the parameters in Table 1 and Table 2. The intermediate growth phase (95%–5% proliferating cells) is indicated as grey bar.

Assessing the impact of a GLUT modulation towards a flux of 6 mmol L^−1^ min^−1^, for instance by an overexpression of GLUT or through the hypoxia-inducible factor 1 [25], [26], in combination with the measurement of ATP, PPP metabolites and NADPH production rates poses a very challenging experimental task. Therefore, the predictive power of the developed model was evaluated by performing a cultivation with a similar medium but with low initial GLC^x^ concentration of 2.5 mmol L^−1^ min^−1^. After adjusting the cell growth model such that it reflects the growth of the cells under low glucose concentrations (i.e. growth depends on glutamine, 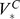 is reduced, and the macroscopic uptake rates depend also on the glucose concentration; see supporting information 4, Fig. S2), the model for glycolysis predicts changes in the peaks of the metabolite pools and a transient shift into a limitation scenario (Fig. 8). The peak in metabolite pools of upper glycolysis as well as R5P and UGLC (see supporting information 4, Fig. S3) is correctly predicted especially with respect to its width. However, the maximum peak height of F6P, F16BP as well as R5P and UGLC exceeds that of the model prediction and is also higher than during the CULT1–3 experiments. At later times of cultivation, the levels of many metabolite pools are low which is similarly predicted by the model. Most interestingly, the model prediction renders the negative peak of 3PG at 48 h of cultivation as well as the very high final level of PEP (Fig. 8).

## Discussion

### Model structure

We developed a kinetic description of glycolysis that, coupled to a segregated cell growth model, enabled describing and analyzing the experimental data of this study comprising roughly 600 data points by using a single set of parameters for the enzyme kinetics. To describe the dynamics of enzyme activities different types of kinetics with arbitrary complexity can be found in literature. Here, we focused on the establishment of a relatively simple model, which incorporates only basic regulatory mechanisms of glycolytic enzymes and a minimum of reactions. Nevertheless, the model reflects the basic dynamics of metabolite pools for a variety of experimental data sets and time scales. In the model, the kinetics of TATK as well as the ENO represent lumped reactions and were realized with reversible mass-action kinetics (see supporting information 3 for further details on enzyme kinetics). The enzymes HK, GPI, G6PDH, UT, and aldolase (ALD) as well as the GLUT were defined as Michaelis-Menten kinetics, as they provide an upper activity bound that was measured *in vitro* by Janke et al. [19] (except GLUT), and appear either as reversible or irreversible reaction. So similarly to mass action kinetics, only one or two parameters of the Michaelis-Menten kinetics required estimation. Only the PFK, which is a strongly regulated enzyme in glycolysis, as well as the PK were considered to be influenced by allosteric effectors. A Hill-Kinetic with four subunits [10], [27] was sufficient for the PFK to fit all data and takes a direct activation by F6P [17] and an indirect activation via fructose 2,6-bisphosphate (F26BP) into account [28], [29]. The PK is influenced by the well-known F16BP-mediated activation. The chosen simplifications in enzyme kinetics renders the used parameters to be more abstract, such that, for example, the affinity of an enzyme for its substrates or products rather represents a constant sum of influential factors such as availability of cofactors and concentration of ions. As a result, a comparatively simple model is obtained that describes the experimental data with enzyme kinetics comprising only 19 parameters. In addition, two experiment-specific parameters were determined for each cultivation, which yields a total of 21 degrees of freedom not considering the parameters used in the segregated cell growth model. In principle, however, any model of glycolysis that takes into account the metabolites and enzyme reactions used here (even though with higher complexity) may equally well describe the dynamics of the intracellular metabolite pools of this study. Nevertheless, our relatively simple model features the identification of mechanisms that are involved in certain dynamics and has the advantage of efficient parameter estimation and model analyses. Furthermore, extension by additional reaction mechanisms is relatively easy in case further experimental data is available or other cellular functions are of interest, e.g. the response of primary metabolism to osmotic stress [30], and hypoxia [31] or its influence on the glycosylation of proteins [32].

### Model coupling and simulation

The derived kinetic description of glycolysis simultaneously integrates data of three independent cell cultivation experiments, two limitation experiments and one pulse experiment and therefore required coupling to a model that takes explicitly into account the progress of the cell through different growth phases during the cultivation experiments Cult1–3 [23]. Because of the many different experimental settings, simulations would normally require a large set of initial conditions that comprise not only starting concentrations of intracellular metabolites (8 degrees of freedom) but also cultivation conditions (the actual medium volume, glucose concentration), and the growth status of the cells (cell number, cell-specific volume, enzyme level and glucose uptake rate). Considering that the perturbation experiments were performed at a certain time point of cultivation and that cultivations in turn were inoculated with cells from a defined preculture introduces a dependency of the cell status on the cultivation history. Accordingly, we transfer information regarding the cell status, which comprises information of growth and metabolism, as well as culture conditions (Table 1) from one simulation to another (Fig. S4, further explained in the supporting information 5). Estimating a certain cell cultivation history not only eliminates the estimation of initial conditions for glycolysis and the growth status of the cell but also supports consistent data simulation and can be used to evaluate biological variations [33]. However, inconsistent data sets or an unknown cell status (e.g. cell status different to those of Cult1–3) may pose a serious challenge for model fitting. For such scenarios the individual selection of initial conditions might be a better option. In this work, however, the estimation of two experiment-specific parameters, which are the E_level_ for the respective cultivation and t* as starting point for the perturbation experiments, as well as a consistent consideration of all data sets outweighed a perfect data fitting and greatly supported our systems-level analysis of glycolysis.

### Glycolytic activity during substrate perturbation

The simulation of the limitation experiments was started with initial conditions of cells (growth status and a metabolic status) that corresponded to a time point t* of the Cult1 experiment (Table 1). The selection of different time points t* readily explains variations in the initial concentration of intracellular metabolite pools that were found between the Lim1 and Lim2 experiment. The actual limitation was induced by reducing the medium volume to 3
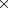
10^−7^ L, which is estimated as liquid volume that remains on the cellular surface or in the intercellular space. In comparison, the volume of all cells is roughly 6
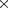
10^−6^ L. In principle, a dilution of the remaining medium with PBS can be realized by choosing lower GLC^x^ concentrations and a higher medium volume (V^M^). The affinity of GLUT for GLC^x^ (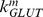) was found to have a large confidence interval and, hence, lower concentrations of GLC^x^ under a higher V^M^ are likewise possible (Table 2).

**Table 2:** Measured and estimated parameters of adherent MDCK cell glycolysis used to simultaneously capture all experiments of this study with confidence intervals between 0.025-quantile 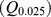 and 0.975-quantile 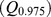.

Parameter	Value	Q_0.025_-Q_0.975_	Unit	Parameter	Value	Q_0.025_-Q_0.975_	Unit
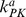	6.56×10^−1^	(5.69–12.09)×10^−1^	mmol^2^ L^−2^	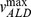	a2.36×10^−11^	-	mmol cell^−1^ min^−1^
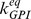	1.78	0.30–10.00^d^	-	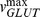	c1.60×10^−11^	-	mmol cell^−1^ min^−1^
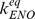	3.80×10^−1^	(2.10–5.19)×10^−1^	-	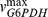	a5.81×10^−11^	-	mmol cell^−1^ min^−1^
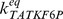	9.84	4.66–969.63^d^	L mmol^−1^	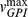	a2.72×10^−10^	-	mmol cell^−1^ min^−1^
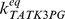	1.01×10^−1^	(0.00–9.31^d^)×10^2^	L mmol^−1^	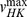	a1.92×10^−11^	-	mmol cell^−1^ min^−1^
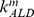	1.77	1.29–2.52	mmol L^−1^	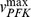	1.00×10^−11^	(0.79–1.15)×10^−11^	mmol cell^−1^ min^−1^
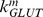	6.60	1.75–35.90	mmol L^−1^	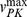	a1.23×10^−9^	-	mmol cell^−1^ min^−1^
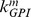	2.41	0.10–2.89	mmol L^−1^	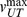	8.17×10^−15^	(6.94–96.81)×10^−15^	mmol cell^−1^ min^−1^
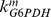	3.98	1.17–10.50	mmol L^−1^	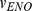	2.34×10^−10^	(2.42–5.81)×10^−10^	mmol cell^−1^ min^−1^
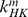	b0.02	-	mmol L^−1^	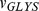	1.91×10^−14^	(1.66–25.89)×10^−14^	L cell^−1^ min^−1^
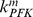	1.08×10^−2^	(0.94–1.38)×10^−2^	mmol L^−1^	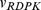	3.69×10^−11^	(1.11–9.49)×10^−11^	L cell^−1^ min^−1^
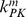	1.66×10^−3^	(0.11–9.99^d^)×10^−3^	mmol L^−1^	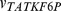	3.73×10^−14^	(3.48–100.20)×10^−14^	L cell^−1^ min^−1^
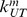	9.96×10^−3^	(1.12–23.24)×10^−3^	mmol L^−1^	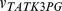	5.60×10^−13^	(0.14–112.22)×10^−13^	L cell^−1^ min^−1^

a value taken from Janke et al. [19],

b value taken from Tsai and Wilson [79],

c value taken from Fitzpatrick et al. [80],

d confidence interval at upper parameter bound.

With the limitation of glycolysis in substrates, the feed-forward regulation of PFK and PK stops the metabolite pool degradation while the TATK reactions partially reverse and fuel glycolysis with 0.03 mmol L^−1^ min^−1^ leading to a new steady state within minutes. Thus, the control of the glycolytic activity shifts from the growth regime that regulates the GLUT activity (see section “Tuning the ATP and biomass precursor generation”) towards an inherent regulation of enzymes by substrates and products in the glycolytic pathway (see also supporting information 1). Without the implementation of the TATK reactions, the remaining glycolytic activity eventually depletes the metabolite pools unless fueled from sources other than GLC. As the limitation applies to all possible extracellular substrates, the use of intracellular carbon sources that might be related to the PPP, glycogenolysis or glyconeogenesis from pyruvate seems likely. The PPP shares already three metabolites with glycolysis (G6P, F6P, and glycerine-aldehyde phosphate linked to 3PG) which are not depleted during the limitation experiments and may thus pose the most promising and simplest option among the aforementioned intracellular carbon sources. Also, the late decrease in R5P during the limitation experiment and its lower level during the pulse experiment may support a scenario in which the PPP fuels glycolysis under limiting GLC levels and, thus, can have a large influence on glycolytic intermediates, which is similarly found for hepatoma cells [34]. In turn, after addition of fresh medium, the PPP metabolite pools may be replenished by glycolysis and we hypothesize a certain buffering capacity of the PPP as it is composed of many reversible reactions and intermediates that participate in the biosynthesis machinery. In the model, the implemented reversible mass action kinetics allow for such a switch from metabolite consumption to metabolite production by the PPP under the lack of alternative sources for glycolysis. However, the flux rates as well as the parameters of the PPP cannot be uniquely identified on the basis of our experimental data (Table 2). Therefore, we have used the additional constrain that the flux from the PPP into glycolysis is low (supporting information 2). Although the implemented mechanisms may not definitely be attributed to the PPP, all parameterizations of Table 2 support the finding that metabolite pools can be maintained (or increased) under limited substrate availability. To this end, the model suggests that the allosteric regulation of PFK and PK as well as the reversibility of GPI and TATK modulate the glycolytic activity in scenarios characterized by limited substrate availability. This is consistent with findings that flux control in glycolysis can rely on a combination of many enzymatic steps [34] and can vary depending on experimental conditions [35]. Counter-intuitively, adenosine-based nucleotides, which are also considered to control the metabolic activity in general [36], are constant during our limitation and pulse experiments (Fig. S5). Similar observations were made for yeast and HeLa cells [17], [37]. Therefore, regulation of glycolytic enzymes of MDCK cells by adenosine-based nucleotides seems unlikely under the conditions investigated, which is also hypothesized by Renner et al. [38] for rat hepatoma cells. Furthermore, an activation of glycolysis by a possibly decreasing ATP/ADP ratio stands in contrast to the metabolite pool preservation and renders its influence to be limited. However, the general purpose of an enzyme-mediated control of the glycolytic activity through PFK, PK, TATK and GPI might lie in the prevention of unnecessary dissipation of valuable biomass precursors and may also guarantee a metabolic status that enables a fast reactivation of glycolysis and other cellular functions when new substrates become available after starvation conditions (Fig. 4C,F,I,L,O).

### Glycolytic activity during cell cultivation

Over the full course of cultivation cells pass through several growth-phases with varying cell-specific volumes and with glucose uptake rates that both strongly influence the metabolite pool dynamics (Fig. 2,3). In addition, abundance of enzymes, their covalent modifications as well as the level of allosteric regulators may change over time which can additionally affect metabolite fluxes and pools [39], [40]. However, to our surprise most of the experimental observations were captured by the model under a parameterization that simultaneously explained the perturbation experiments. Obviously, other hierarchical control mechanisms besides the growth regime (for example on the genome or proteome level) were not essential for describing the observed metabolite pool dynamics. This may be attributed to the fact that initial culture conditions were tightly controlled and that the media composition provided adequate substrate and by-product concentrations in the time span analyzed. Nevertheless, the inclusion of other levels of hierarchical control, in addition to the growth regime of this work, may contribute to simulated aspects of the observed dynamics. The enzyme kinetics and the direct influence by the growth regime are in the following considered as the sole source of regulatory principles that control glycolysis during MDCK cell cultivation.

First, the peak in the metabolite pools can be explained with a high GLUT-mediated flux rate in combination with low cell volume-specific enzyme activities (based on higher cell-specific volumes during the growth phase). The implemented enzyme kinetics realize a relatively higher net flux into the PPP during cell growth, which is attributed to the higher metabolite levels in glycolysis and similarly described by Wu et al. [41] for bovine venular endothelial cells after addition of citrate in order to inhibit the PFK activity. Also, the activation of GLUT in rat thymus lymphocytes with concanavalin A resulted in higher fluxes in glycolysis and into the PPP [42]. Higher fluxes into the PPP possibly enables enhanced nucleotide, macromolecule, and lipid synthesis rates, as reviewed by Mazurek et al. [43]. According to our simulations the fluxes are in the range of 13—15% of the glycolytic flux, which is reasonable for continuously growing mammalian cells in the exponential growth phase [37], [44]. However, a much lower contribution e.g. 5.8% and 3.6% can be found in the late intermediate growth phase, which corresponds to findings for other transformed mammalian cells [45]–[47]. So, the regulation of enzymes by substrates, products and allosteric effectors can change concentrations of intracellular metabolite pools, and reorganize the pathway fluxes, especially under limiting conditions (see section “Glycolytic activity during substrate perturbation”). However, during MDCK cell cultivation the control over the glycolytic activity is exerted by the growth regime through modulation of the GLUT activity. For many microorganisms, the GLUT is described as the rate limiting step that can control the glycolytic flux [38], [48]–[51]. But also adenosine-based nucleotides are reported to play a major role in the control of the glycolytic activity [36], [52]. For MDCK cells, the influence of adenosine-based nucleotides on glycolysis seems to be negligible during cultivation conditions with excess of substrates [22]. So, neither during cell growth nor during substrate perturbation the adenosine-based nucleotides played a crucial role in describing the dynamics of the measured metabolite pools. Therefore, we assumed for the model that enzymes are insensitive against changes in the adenosine-based nucleotide levels, which is also reported by Soboll et al. [53] for rat liver cells.

Snoep and co-workers hypothesized that GLUT controls cell growth [54]. This, however, raises the question, whether metabolism regulates cell growth or vice versa [55]. In case of adherent MDCK cell growth with sufficient substrate supply, the growth status is exclusively defined by the availability of free space on the well surface. Eventually, space becomes limiting and cells reduce the glycolytic activity although high extracellular glucose concentrations are present. Therefore, we hypothesize that the growth regime of exponentially growing MDCK cells controls the GLUT activity to realize a higher metabolic activity yielding in turn higher metabolite pools that meet the energy and precursor demands of the biosynthesis machinery. On a lower level of regulation, the properties of the involved enzymes shape metabolism by influencing flux distributions. Under substrate limitation, however, regulation of enzymes has full control over the glycolytic activity (see section “Glycolytic activity during cell cultivation”). Thus, the model considers that the regulation of the glycolytic activity changes with the physiological status of the cell [55] and sheds light on the regulatory principles that are essential to simultaneously explain various experimental scenarios. Although regulation of glycolysis can change with the microorganism [52], we are convinced that the derived principles can be applied to other metabolic pathways, such as the citric acid cycle [56], and also support the study of other mammalian cell lines relevant for production of biologicals [57].

### Tuning the ATP and biomass precursors generation

Within a GLUT activity of 0–4 mmol L^−1^ min^−1^, the model for glycolysis is validated with cultivation, limitation and pulse experiments. It already shows a good predictive power for an experiment were MDCK cells were grown in DMEM medium with low GLC^x^ levels (Fig. 8), which further strengthens the confidence in the model structure and its parameterization. Although the model prediction for the DMEM cultivation would benefit from a lower E_level_ to describe all maximum peak-heights, it still confirms the close linkage of GLUT activity and intracellular metabolite dynamics. Based on the finding that the GLUT modulates the glycolytic activity during cell cultivation (under sufficient substrate availability) it seemed desirable to explore the maximum capacity of glycolysis and the corresponding ATP and PPP metabolite production. However, such a maximum capacity clearly depends on the enzyme content (E_level_) and the cell-specific volume (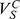). Therefore, we exemplary analyzed cells from the Cult1 experiment at 24 h of cultivation with an actual uptake of 3.3 mmol L^−1^. For these cells, *in silico* modulation of the GLUT activity revealed that an uptake of up to 3.8 mmol L^−1^ min^−1^ can be realized until the glycolytic flux saturates the PFK capacity, which slightly enhances the ATP production on average to 105%, and the PPP metabolite and NADPH production to surprising 153% for cells of Cult1 at 24 h. According to the model, a further increase in ATP production would require the simultaneous overexpression of the PFK, which illustrates the difficulty in fast up-regulation of metabolic activity while keeping a certain balance between ATP and PPP metabolite production. However, Janke et al. [19] measured higher maximum *in vitro* PFK activities than estimated in this study and glycolysis of MDCK cells may have higher capacities than estimated by the model. Higher biomass precursor and ATP production rates can support higher growth rates as shown for tumor and yeast cells with up-regulation of the GLUT activity [58], [59]. Furthermore, Schmidt et al. [60] described a correlation between the growth of tumor cells and the ATP production rate. Potentially, an increase in the ATP production to 105% may not or only slightly support higher growth rates for MDCK cells especially as they are described to have a large overproduction in ATP [47], [61]. But due to the importance of PPP metabolite production to pyrimidine [43], [62] and purine production [63] and NAPDH to lipid synthesis we believe that an increase to 153% positively affects the growth of cells (Fig. 6). A glycolytic activity above 5 mmol L^−1^ min^−1^ drastically enhances the production of PPP metabolites (433%) at the expense of the ATP production (77%) and seems to be an interesting scenario for future experiments. However, also the reduction in the glucose uptake, as done by Liebl et al. [64], poses an interesting strategy to design a more economic breakdown of glucose in biotechnological processes [65]. Currently, the reduction of the glucose uptake by interference with the glucose transporter is also studied as a potential target for cancer treatment [59], [66] which may benefit from the acquisition of mathematical models to evaluate corresponding dynamics in metabolism. Taken together, the model can greatly support the development of strategies that aim either at a faster or a more efficient cell growth, and is also an aid in the design of new experiments.

## Materials and Methods

### Model and simulation

The differential algebraic equations of the glycolytic model were composed of first order rate laws, Michaelis-Menten and Hill kinetics which describe enzyme activity in dependence of metabolite concentrations and allosteric influences.

#### Coupling of glycolysis to a segregated cell growth model

To simulate intracellular metabolite dynamics during cell cultivation the kinetic description of glycolysis was coupled to the recently developed segregated cell growth model of Rehberg et al. [23]. In principle, any cell growth model can be used for the coupling as long as it provides information regarding the glucose uptake rate and the changes in mean cell diameter. To facilitate simulations and to allow for model analyses (i.e. parameter sensitivity studies) the cell growth model should be simple and only incorporate state variables available from experiments. Coupling of models incorporating delay functions (e.g. [67]) or population balance equations (e.g. [68]) will involve significant challenges regarding efficiency of algorithms and time required for simulations. Therefore, we specifically developed a segregated cell growth model for the coupling to kinetic descriptions of metabolic pathways. Limitations and advantages of the model are in detail described by Rehberg et al. [23].

The segregated growth model established [23], provides all information regarding the medium volume-specific uptake rate of GLC^x^ for growth (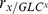) and for maintenance (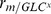), the cell-specific volume (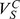), the specific growth rate (*μ*), and a cell volume-dependent growth inhibition factor (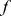) that increases over cultivation time (see supporting information 6 for further specification of parameters of the segregated cell growth model). Furthermore, the water evaporation constant (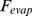), medium volume (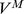), and cell number (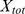) are taken into account. The concentration of 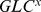 decreases over cultivation time with
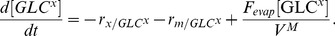
(1)


For the simulation of the perturbation experiments we assumed that the glucose uptake depends on a variable capacity for glucose trans-membrane transport (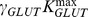) as well as on the affinity of GLUT for 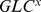
(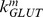) such that Eq. (1) is substituted by
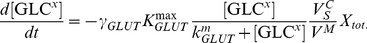
(2)


The variable 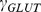 scales the glucose uptake kinetic to the macroscopic description in Eq. (1) at any time point of cultivation at which the perturbation experiment starts (t*; Table 1). Hence, 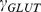 stands for cellular mechanisms such as a change in glucose affinity of GLUT, translocation of GLUT or molecule-based activation of GLUT [69] that are necessary to meet the rate in Eq. (1):
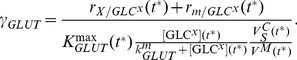
(3)

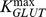
 is the cell volume-specific maximum transport activity of GLUT that is calculated from the cell-specific maximum transport activity 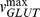, the 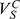 and the 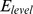:
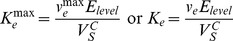
(4)


Note that *e* stands for GLUT or any other enzyme of the model and 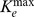 is, hence, the maximum cell volume-specific activity for enzyme *e*. For first order rate laws, the cell volume-specific enzyme activity 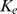 is similarly derived from the cell number-specific activity 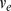.

#### Kinetics of glycolytic enzyme reactions

The model of glycolysis considers the metabolic conversion of GLC to PYR as well as the interconnection with PPP and glycogenesis for an average cell:
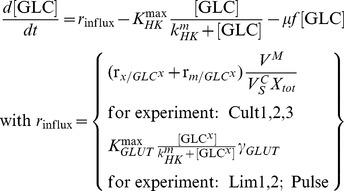
(5)

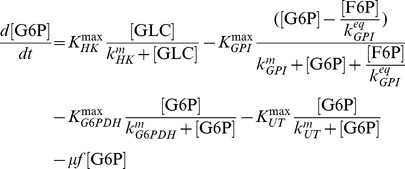
(6)

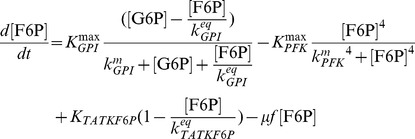
(7)

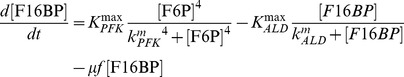
(8)

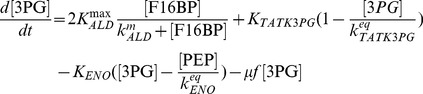
(9)

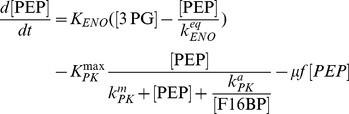
(10)


The term 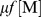 expresses the dilution of intracellular metabolite M by the approximate cell volume growth (which is assumed to be zero during the perturbation experiments) while the used parameters were defined as follows: 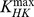, 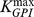, 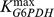, 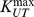, 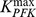, 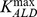, and 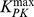 are the maximum cell volume-specific enzyme activities of HK, GPI, G6PDH, UT, PFK, ALD, and PK, respectively; 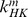, 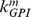, 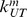, 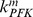, 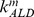, and 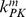 are the affinity constants of HK for GLC, of GPI for G6P, of UT for G6P, of PFK for F6P, of ALD for F16BP, and of PK for PEP, respectively; 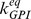, 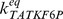, 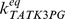, 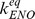 are the equilibrium constants of GPI between G6P and F6P, of TATKF6P between F6P and the PPP pool (can have an arbitrary constant level; here 1 mmol L^−1^), of TATK3PG between 3PG and the PPP pool, and of ENO between 3PG and PEP; 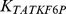, 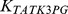, and 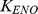 are the cell volume-specific enzyme activities of TATK with respect to F6P conversion, TATK with respect to 3PG conversion, and ENO. 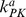 is the activation constant of PK by F16BP.

#### Pentose phosphate pathway and glycogenesis

The model of glycolysis was extended at the expense of two additional parameters by R5P and UGLC:
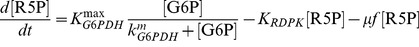
(11)

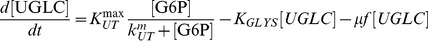
(12)


The ribose-phosphate diphosphokinase and glycogen synthase with activity 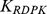 (Eq.(11)) and 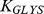 (Eq.(12)), respectively, were modeled using first order rate laws. Additional constraints for metabolite exchange between glycolysis and the PPP are described in the supporting information 2.

#### Simulation procedure, initial values and parameter settings

For simulation of the batch cultivation experiments Cult1–3, the kinetic description of glycolysis is coupled to the segregated cell growth and thus requires initial conditions for the growth status of the cell as well as the culture conditions, which are given by Rehberg et al. [23] (same symbol and color code). However, the initial conditions for the metabolic status were derived by simulating Cult1 at 200 h of cultivation for longer times (10^4^ min). The E_level_ was estimated for each cultivation experiment. For simulation of the perturbation experiments, the actual growth status and the metabolic status of cells at time point t* of Cult1 was used. Therefore, in addition to the estimation of 19 kinetic parameters, two experiment-specific parameters (t* and E_level_) need to be estimated (see also Table 1 and Table 2). Furthermore, it was assumed for the perturbation experiments that cells remain constant in size and number. Substrate limitation was initiated by reducing the medium volume 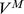 to 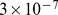 L, which considers remaining glucose at the cellular surface and in the inter-cellular space. Simulation of the pulse experiment was initialized with the metabolic and cell growth status present after two hours of limitation and the culture conditions 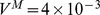 L and 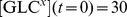 mmol L^−1^. An overview of all initial conditions and settings is given in Table 1. A flow sheet for the transfer of initial conditions and experiment-specific parameter to the corresponding routines is given in Fig. S4 (also see the supporting information 5).

#### Computation

For model fitting, estimation of parameter confidence intervals, and visualization of results MATLAB (Version R2012b, The MathWorks, Inc.) was used. Models and data were handled with the Systems Biology Toolbox 2 developed by Schmidt and Jirstrand [70]; the model is exemplary given as .txt file for simulation of Cult1 (Model S1) and Lim1 (Model S2), the kinetic description of glycolysis is also provided in the SBML format (level 2 version 4, Model S3). Integrations of the ordinary differential equations were performed with the CVODE from SUNDIALS [71]. The algorithm SSm [72] was used for stochastic global optimization of the parameters and experiment settings using a least squares objective function, which considers the constraints given in the supporting information 2. A bootstrap method [73], [74] was used for assessment of the parameter confidence intervals with a total of 2000 runs. All simulations were carried out on a Linux-based system.

### Experiments and analytics

#### Cell cultivation

As already described by Rehberg et al. [22], Madin Darby Canine Kidney (MDCK) cells (ECACC, #84121903) were precultured in GMEM (Gibco, #22100-093), supplemented with 10% fetal calf serum (Gibco, #10270-106), 2 g L^−1^ peptone (International Diagnostics Group, #MC33) and 4 g L^−1^ NaHCO3 (Roth, #6885.1), referred to as GMEM-Z. Precultures were either carried out in roller bottles (Greiner Bio-One, #680XX, experiment depicted in figures with symbol ○) or in T-flasks (Greiner Bio-One, #661160, experiments depicted in figures with symbols ▵ and □) at 37°C and 5% CO_2_. Cell cultivation experiments Cult1 (▵), Cult2 (□) and Cult3 (○) were independently performed in parallel 6-well plates (Greiner Bio-One, #657160) containing 4 mL GMEM-Z with an average initial cell concentration of about 6×10^5^ cells well^−1^, cultivated at 37°C and 5% CO_2_ in an incubator. Subsequent analytics were applied to at least three individual wells per time point. For the perturbation experiments, cells were grown to the late exponential growth phase at which a sample was taken as time point zero. Glucose limitation was achieved by discarding the medium followed by an immediate washing step with PBS (8 g L^−1^ NaCl, 0.2 g L^−1^ KCl, 0.2 g L^−1^ KH_2_PO_4_, 1.2 g L^−1^ Na_2_HPO_4_) and followed by addition of PBS. After 2 h of limitation, a sample was taken as time point zero of the glucose pulse experiment where PBS was removed and 4 mL of fresh GMEM-Z added. Additionally, MDCK cells from a GMEM-Z preculture were inoculated with a concentration of 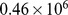 cells per well in 4 mL DMEM medium (#E15-079, PAA Laboratories), which was supplemented with 10% fetal calf serum (Gibco, #10270-106), 2 g L^−1^ peptone (International Diagnostics Group, #MC33) as well as 2.5 mmol L^−1^ glucose and 2.0 mmol L^−1^ glutamine.

#### Analytics

The applied analytics are in detail described in [75]. In short, after removal of the supernatant, cells were washed three times with PBS and treated 30 min with trypsin (2.5%, porcine, 5 U, 0.5 mL per well, Gibco, #27250-018) for cell detachment. Cells were harvested using a cell scraper. A Vi-Cell TM XR Cell Viability Analyzer (Beckman Coulter) was used for cell counting and measurement of the diameter distribution. Cell number and cell diameter distribution were used to determine the cell volume. GLC^x^ concentrations in the supernatant were quantified as described by Genzel and Reichl [76] using a Bioprofile 100 plus analyzer (Nova Biomedical, relative standard deviation of the method 1.9–6.4% [77]). For the measurement of intracellular metabolites, sample preparation was performed as described in detail by Ritter et al. [75] using ice cold solutions. The medium of the wells was discarded and the cell layer was washed with a 0.9% NaCl solution. Quenching of metabolic reactions and extraction of metabolites was done by immediate addition of MeOH/CHCL_3_ solution (1∶1). Quantification of the intracellular metabolites was performed by anion exchange chromatography (BioLC system, Dionex) in combination with mass spectrometry (LC-MS, relative standard deviation of the method 0.7–9.5%), as described by Ritter et al. [20] and Ritter et al. [78]. The absolute amount of metabolites per well were related to the measured cell volume at respective times of cultivation. To reduce the error, regression analysis was used to interpolate the measured cell volume. The limit of quantification was related to the simulated cell volume 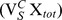.

## Supporting Information

Figure S1 **Sensitivity analysis of initial conditions and model parameters.** (**A**) Relative local sensitivity of model simulations (for cultivation and perturbation experiments) to a 1% perturbation in glycolysis parameters (Table 2), parameters of the segregated cell growth model (Rehberg et al., 2013), culture conditions growth status and metabolic status (Table 1). (**B**) Relative local sensitivity of model simulations (for cultivation and perturbation experiments) to a 1% perturbation of single glycolysis parameter.
(TIF)

Figure S2 **Adjusting the segregated growth model established previously** **[14] to MDCK cell proliferation in 6-well plates using DMEM medium.** Cell number (**A**), mean cell diameter (**B**) and extracellular glucose concentration (**C**) during MDCK cell cultivations in 6-well plates and DMEM medium with 2.5 mmol L^−1^ extracellular glucose. Data (◊) and error bars represent mean and standard deviation of three wells. Lines represent the respective simulation result based on the modifications described in supporting information 4. The intermediate growth phase (95%–5% proliferating cells) is indicated as grey bar.
(TIF)

Figure S3 **Prediction of ribose 5-phosphate and uridyl diphosphate glucose during cultivation of MDCK cells in DMEM with limited extracellular glucose.** Ribose 5-phosphate (**A**) and uridyl diphosphate glucose (**B**) concentrations during MDCK cell cultivations in 6-well plates and DMEM medium with 3 mmol L^−1^ extracellular glucose. Data (◊) and error bars represent mean and standard deviation of three wells. Dashed lines are the limit of quantification (LOQ; data below LOQ marked in grey). Lines represent the respective simulation result based on the parameters of Table 1 and experiment-specific parameters of Table 2. The intermediate growth phase (95%–5% proliferating cells) is indicated as grey bar.
(TIF)

Figure S4 **Flow of information and link of experimental data.** 1) Transfer of growth status and culture condition occurring in Cult1 at 200 h of cultivation to determine the metabolic status by steady state simulation. 2) Transfer of the metabolic steady state to the simulation of the Cult1–3 and the Pred. simulation. 3) At individual time points t*, the metabolic and growth status of Cult1 is transferred to the respective simulation of the Lim1–3 experiments. 4) Simulation of pulse response with initial conditions determined with the Lim3 simulation. Green background: Coupling of segregated cell growth model and structured model of glycolysis; red background: coupling of adjusted segregated cell growth model, which renders cell growth under limited GLC^x^ concentrations, to the structured model of glycolysis.
(TIF)

Figure S5 **Adenosine-based nucleotide pools during perturbation experiments.** ATP (**A**–**C**), ADP (**D**–**F**) and AMP (**G**–**I**) concentrations in three independent perturbation experiments with MDCK cells in 6-well plates. Cells, originating from a cultivation experiment, are limited in extracellular nutrients by removal of medium and addition of phosphate buffered saline (PBS), shown in the first column (Lim1, **A**,**D**,**G**) and second column (Lim2, **B**,**E**,**H**). After two hours of incubation, PBS was exchanged by fresh medium (Pulse, **C**,**F**,**I**). Data (○) and error bars represent mean and standard deviation of three wells while dashed lines are the limit of quantification.
(TIF)

File S1 **SBML model for yeast glycolysis adapted to simulate a glucose limitation scenario.**
(XML)

Model S1 **Segregated cell growth model coupled to the structured model of glycolysis for simulation of Cult1.** The model is provided as .txt and can be computed with the Systems Biology Toolbox 2 (see section “Computation”).
(TXT)

Model S2 **Structured model of glycolysis for simulation of Lim1.** The model is provided as .txt and can be computed with the Systems Biology Toolbox 2 (see section “Computation”).
(TXT)

Model S3 **Structured model of glycolysis for simulation of Lim1.** The model is provided in the SBML format level 2 version 4.
(XML)

Supporting Information S1 **Sensitivity analysis of initial conditions and model parameters.**
(DOCX)

Supporting Information S2 **Constraints for metabolite exchange with the PPP.**
(DOCX)

Supporting Information S3 **Detailed description of enzyme kinetics.**
(DOCX)

Supporting Information S4 **Predicting the glycolytic activity during cell growth in DMEM medium.**
(DOCX)

Supporting Information S5 **Flow of information and initial conditions for parameter fitting.**
(DOCX)

Supporting Information S6 **Nomenclature for parameter of the segregated cell growth model.**
(DOCX)
