# Supplementary figures and images for: Glycolysis Is Governed by Growth Regime and Simple Enzyme Regulation in Adherent MDCK Cells
Source: PLoS Comput Biol. 2014 Oct 16;10(10):e1003885. doi: 10.1371/journal.pcbi.1003885 (PMC4211564; doi:10.1371/journal.pcbi.1003885)

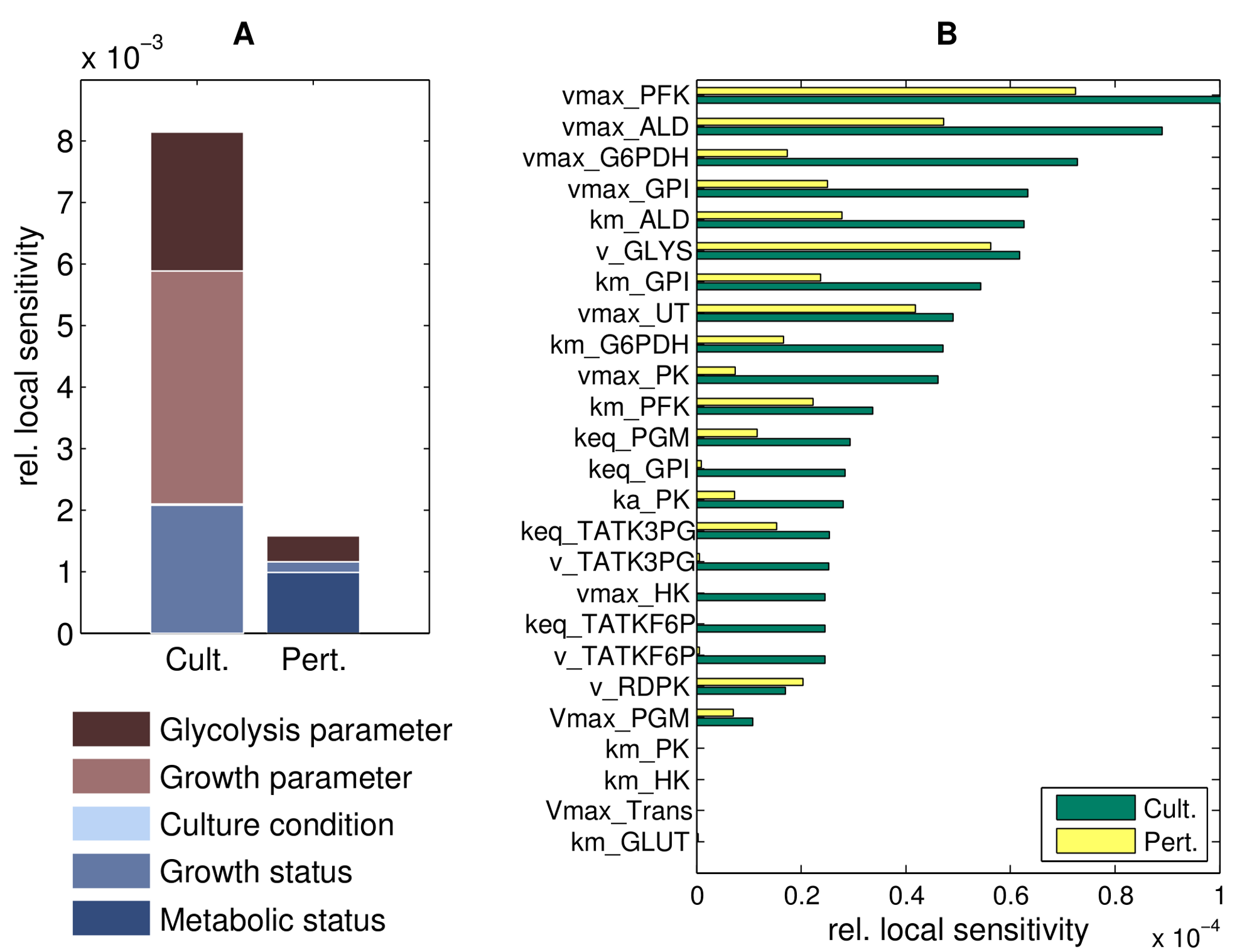

Supplement: Figure S1 — Sensitivity analysis of initial conditions and model parameters. (A) Relative local sensitivity of model simulations (for cultivation and perturbation experiments) to a 1% perturbation in glycolysis parameters (Table 2), parameters of the segregated cell growth model (Rehberg et al., 2013), culture conditions growth status and metabolic status (Table 1). (B) Relative local sensitivity of model simulations (for cultivation and perturbation experiments) to a 1% perturbation of single glycolysis parameter. (TIF) [file pcbi.1003885.s001.tif]

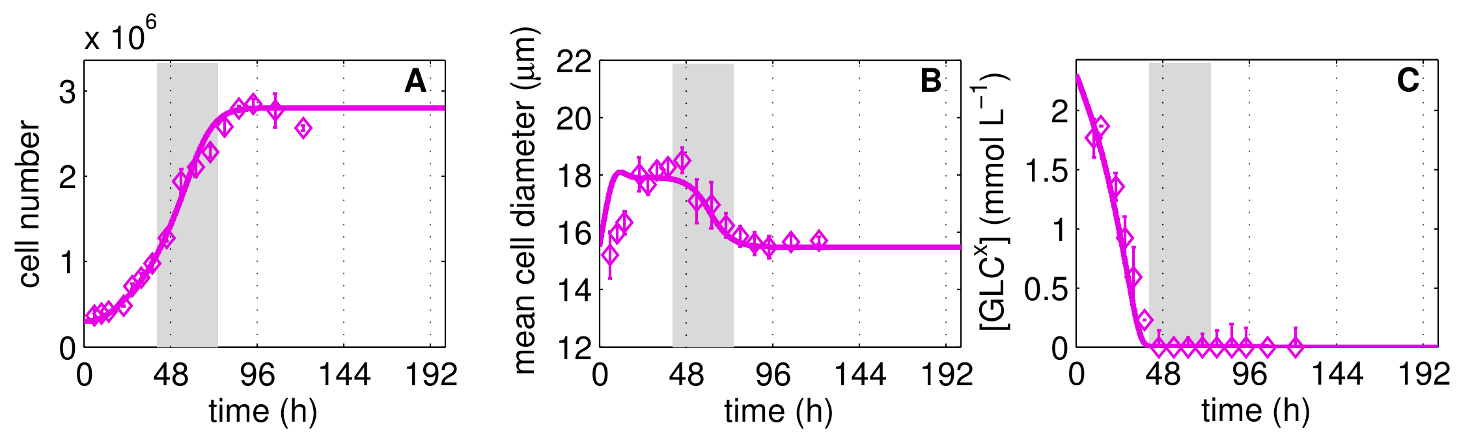

Supplement: Figure S2 — Adjusting the segregated growth model established previously [14] to MDCK cell proliferation in 6-well plates using DMEM medium. Cell number (A), mean cell diameter (B) and extracellular glucose concentration (C) during MDCK cell cultivations in 6-well plates and DMEM medium with 2.5 mmol L−1 extracellular glucose. Data (◊) and error bars represent mean and standard deviation of three wells. Lines represent the respective simulation result based on the modifications described in supporting information 4. The intermediate growth phase (95%–5% proliferating cells) is indicated as grey bar. (TIF) [file pcbi.1003885.s002.tif]

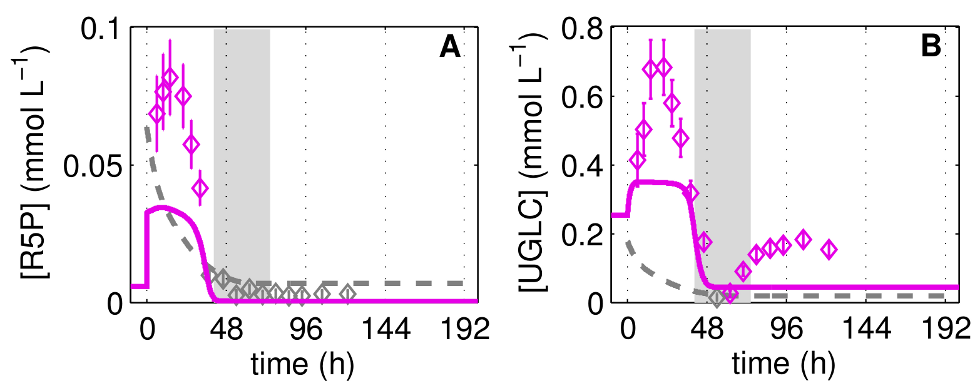

Supplement: Figure S3 — Prediction of ribose 5-phosphate and uridyl diphosphate glucose during cultivation of MDCK cells in DMEM with limited extracellular glucose. Ribose 5-phosphate (A) and uridyl diphosphate glucose (B) concentrations during MDCK cell cultivations in 6-well plates and DMEM medium with 3 mmol L−1 extracellular glucose. Data (◊) and error bars represent mean and standard deviation of three wells. Dashed lines are the limit of quantification (LOQ; data below LOQ marked in grey). Lines represent the respective simulation result based on the parameters of Table 1 and experiment-specific parameters of Table 2. The intermediate growth phase (95%–5% proliferating cells) is indicated as grey bar. (TIF) [file pcbi.1003885.s003.tif]

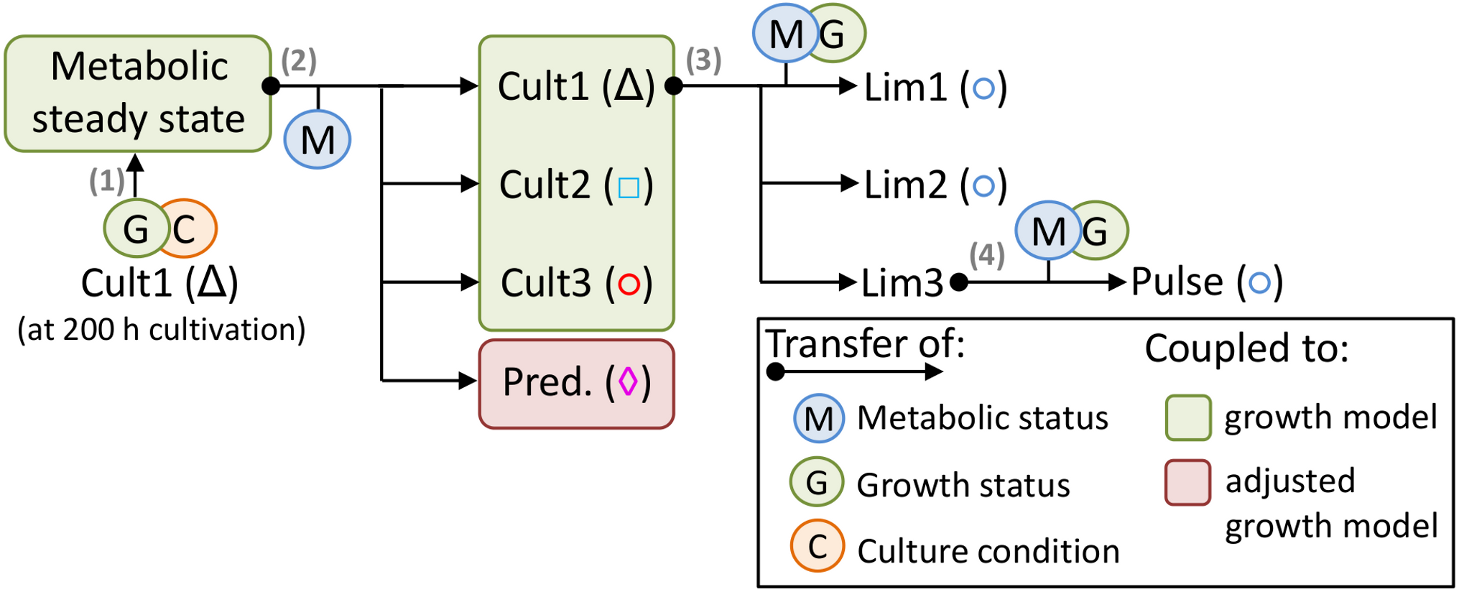

Supplement: Figure S4 — Flow of information and link of experimental data. 1) Transfer of growth status and culture condition occurring in Cult1 at 200 h of cultivation to determine the metabolic status by steady state simulation. 2) Transfer of the metabolic steady state to the simulation of the Cult1–3 and the Pred. simulation. 3) At individual time points t*, the metabolic and growth status of Cult1 is transferred to the respective simulation of the Lim1–3 experiments. 4) Simulation of pulse response with initial conditions determined with the Lim3 simulation. Green background: Coupling of segregated cell growth model and structured model of glycolysis; red background: coupling of adjusted segregated cell growth model, which renders cell growth under limited GLCx concentrations, to the structured model of glycolysis. (TIF) [file pcbi.1003885.s004.tif]

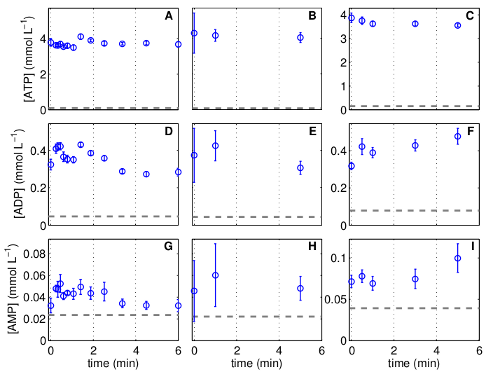

Supplement: Figure S5 — Adenosine-based nucleotide pools during perturbation experiments. ATP (A–C), ADP (D–F) and AMP (G–I) concentrations in three independent perturbation experiments with MDCK cells in 6-well plates. Cells, originating from a cultivation experiment, are limited in extracellular nutrients by removal of medium and addition of phosphate buffered saline (PBS), shown in the first column (Lim1, A,D,G) and second column (Lim2, B,E,H). After two hours of incubation, PBS was exchanged by fresh medium (Pulse, C,F,I). Data (○) and error bars represent mean and standard deviation of three wells while dashed lines are the limit of quantification. (TIF) [file pcbi.1003885.s005.tif]
