# Supplementary material for: Glycolysis Is Governed by Growth Regime and Simple Enzyme Regulation in Adherent MDCK Cells
Source: PLoS Comput Biol. 2014 Oct 16;10(10):e1003885. doi: 10.1371/journal.pcbi.1003885 (PMC4211564; doi:10.1371/journal.pcbi.1003885)
Supplement: Supporting Information S1 — Sensitivity analysis of initial conditions and model parameters. (DOCX) [file pcbi.1003885.s010.docx]

# Supporting information 1: sensitivity analysis

We applied a sensitivity analysis to assess the influence of the parameters of the structured model of glycolysis (Table II), the parameters of the segregated cell growth model [1], the culture conditions, the growth status, and the metabolic status (Table I) on the model behavior. According to Gutenkunst *et al.* [2], the change in model behavior ($\chi^{2}$) over all experiments$l=1,...,N^{l}$, measured metabolite pools $m=1,...,N_{j}^{m}$ and time points $j=1,...,N_{lm}^{j}$ in response to a change in parameter or initial conditions ($\Theta$) is described with:

| $\chi^{2}(\Theta)=\frac{1}{2}\sum_{l=1}^{N^{l}} \sum_{m=1}^{N_{l}^{m}} \sum_{j=1}^{N_{lm}^{j}} \frac{1}{N_{l}^{m}N_{lm}^{j}}\left(\frac{y_{lmj}(\Theta)-\mathrm{y}_{lmj}(\Theta')}{\sigma^{2}}\right)$ | (1) |
| --- | --- |

with $y(\Theta)$ being the matrix of model trajectories and $\sigma^{2}=\max_{lm}([\mathrm{y}_{lm1}(\Theta),...,\mathrm{y}_{lmN_{lm}^{j}}(\Theta)])$ being a normalization for metabolite pool *m* in experiment *l*. For the sensitivity analysis a 1 % change in a single parameter value of $\Theta$ was used, with $\Theta'$ being the perturbed set of parameters or initial conditions.

Fig. S5A (Cult) shows that the simulations of the cell cultivation experiments are sensitive to the glycolytic parameters, the growth parameters (of the segregated cell growth model) and the growth status. In contrast, the metabolic status of cells has no influence on the model behavior, even if the perturbation is increased from +1 % to $\pm$20 %, which can be explained with a high glycolytic activity and low metabolite pool sizes (see main text, section 2.1.1). In agreement to our previous analysis of MDCK cells grown in two different media [3], the culture conditions (GLC^x^, V^M^) have only a marginal influence on the model behavior. However, with onset of a substrate limitation the simulations become sensitive to the metabolic status of cells (Fig. S5A, Pert), while the growth status and the glycolysis parameter still have a significant influence on the model behavior. The culture conditions have almost no influence and the model is also insensitive against changes in the growth parameters, which is expected. The overall reduction in the sensitivities is attributed to the simulation scenario. The simulation of a limitation experiment yields more robust results than the simulation of a cultivation experiments upon a variation of 1 % in $\Theta$.Taken together, the sensitivity analysis supports our hypothesis that the control of glycolysis shifts from the growth regime that modulates the GLUT to a coordinated control by glycolytic enzymes. A deeper analysis of the dynamics of the structured model of glycolysis reveals $v_{PFK}^{\max}$ as the most influential parameter of glycolysis during cultivation and perturbation experiments as the phosphofructokinase (PFK) controls the degradation of metabolite pools of upper glycolysis (Fig. S5B). However, most of the remaining parameters of the structured model of glycolysis also have a significant influence on the model behavior. That slight changes in the parameters of the kinetics for HK and GLUT have no impact on simulation results is discussed in section 3.3 of the main text, and in the supplementary information 2, respectively. The PK reaction is very sensitive to PEP levels and therefore insensitive against slight changes in $k_{PK}^{m}$.

# References

1. Rehberg M, Ritter JB, Genzel Y, Flockerzi D, Reichl U (2013) The relation between growth phases, cell volume changes and metabolism of adherent cells during cultivation. J Biotechnol.

2. Ryan N. Gutenkunst, Joshua J. Waterfall, Fergal P. Casey, Kevin S. Brown,Christopher R. Myers, and James P. Sethna. Universally sloppy parameter sensitivities in systems biology models. PLoS Comput Biol, 3(10):1871-1878, Oct 2007. doi: 10.1371/journal.pcbi.0030189.

3. Rehberg M, Rath A, Ritter JB, Genzel Y, Reichl U (2013) Changes in intracellular metabolite pools during growth of adherent MDCK cells in two diffferent media. Appl Mircobiol Biotechnol.
