# Supplementary material for: Glycolysis Is Governed by Growth Regime and Simple Enzyme Regulation in Adherent MDCK Cells
Source: PLoS Comput Biol. 2014 Oct 16;10(10):e1003885. doi: 10.1371/journal.pcbi.1003885 (PMC4211564; doi:10.1371/journal.pcbi.1003885)
Supplement: Supporting Information S2 — Constraints for metabolite exchange with the PPP. (DOCX) [file pcbi.1003885.s011.docx]

# Supporting information 2: constraints for metabolite exchange with the PPP

Additional constraints for metabolite exchange with the PPP were considered during parameter estimation. Firstly, the net flux from glycolysis into the PPP is considered to be in a typical biological range of 0 % to 40 % of the glycolytic activity, which was taken into account as the following constraint:

$$0<\frac{(\mathrm{G}6\mathrm{PDH}-\mathrm{TATKF}6\mathrm{P}-0.5\cdot\text{TATK3PG})}{\text{HK}}<0.4$$

Secondly, during glucose limitation, the pentose phosphate pathway can fuel glycolysis but presumably at a very low rate. Therefore, the activity of the pyruvate kinase was constraint at the end of the limitation experiments to be in maximum 0.04 mmol L^-1^ min^-1^ (which corresponds to a 3 % activity of glycolysis in the stationary growth phase):

$$PK(t=6\min)<0.04\text{ mmol L}^{-1}\min^{-1}$$
