# Supplementary material for: Glycolysis Is Governed by Growth Regime and Simple Enzyme Regulation in Adherent MDCK Cells
Source: PLoS Comput Biol. 2014 Oct 16;10(10):e1003885. doi: 10.1371/journal.pcbi.1003885 (PMC4211564; doi:10.1371/journal.pcbi.1003885)
Supplement: Supporting Information S3 — Detailed description of enzyme kinetics. (DOCX) [file pcbi.1003885.s012.docx]

# Supporting information 3: detailed description of enzyme kinetics

**Glucose transporter (GLUT):** the glucose transport kinetic was defined as Michaelis-Menten kinetic, as already suggested by [Luni et al (2012)](#_ENREF_6) for pancreatic β-cells. Because of the multiplication of $v_{GLUT}^{\max}$ with $\gamma_{GLUT}$, an estimation of $v_{GLUT}^{\max}$ is not possible in this work and was chosen to be $1.60\times10^{-11}$ which agrees with findings for other mammalian cell lines ([Fitzpatrick et al, 1993](#_ENREF_2); [Rivenzon-Segal et al, 2000](#_ENREF_11)).

**Hexokinase (HK, EC number: 2.7.1.1)**: firstly, GLC could not be detected experimentally in MDCK cells, which was similarly reported for other cell lines ([Renner et al, 1972](#_ENREF_10); [Schmid & Blanch, 1992](#_ENREF_12)). Therefore, a possible regulation by ATP, as measured by [Fromm and Zewe (1962)](#_ENREF_3) for yeast cells, may impair the hexokinase activity but presumably not to a degree sufficient to limit the glycolytic activity. Due to the lack of appropriate data for GLC and the lack of any indication regarding a possible regulation by ATP we assumed a simple Michaelis-Menten kinetic, which considers the maximum *in vitro* enzyme activity measured by [Janke et al (2011)](#_ENREF_4) for MDCK cells and an affinity constant for glucose of 0.02 mmol L^-1^ ([Neermann & Wagner, 1996](#_ENREF_7)), which together yield a highly active HK and low GLC levels.

**Glucosephosphate isomerase (GPI, EC number: 5.3.1.9):** a reversible Michaelis-Menten kinetic was used to describe the isomerization of G6P and F6P as the maximum *in vitro* activity was measured by [Janke et al (2011)](#_ENREF_4).

**Glucose 6-phosphate dehydrogenase (G6PDH, lumped reaction):** the G6PDH reaction of this work represents the conversion of G6P to R5P, which produces also CO_2_ and NADPH. It is implemented as simple Michaelis-Menten kinetic as the maximum *in vitro* activity was measured by [Janke et al (2011)](#_ENREF_4).

**Ribose 1,5-bisphosphate phosphokinase (RDPK, EC number: 2.7.4.23):** to illustrate the degradation of R5P, e.g. for biosynthesis, the RDPK is implemented as a first order rate law. However, R5P can also be converted to F6P and 3PG. Therefore, the effective degradation rate of pentose phosphate metabolites is only indirectly given as $G6PDH-TATKF6P-0.5\cdot TATK3PG$.

**Uridyl transferase (UT, lumped reaction):** in glycogenesis UT is involved in the generation of UGLC. A Michaelis-Menten kinetic close to saturation was required to describe the experimental data.

**Glycogen synthase (GLYS, EC number: 2.4.1.11):** UGLC is degraded either for the synthesis of glycogen or other cellular needs, which was considered by using a first order rate law.

**Phosphofructo kinase (PFK, EC number: 2.7.1.11):** the phosphofructo kinase is a highly regulated enzyme in glycolysis and often considered to be best represented by kinetics that consider a certain cooperativity. In agreement to [Otto et al (1977)](#_ENREF_8) and [Boiteux and Hess (1981)](#_ENREF_1) we considered a cooperativity of n = 4. A very potent regulator of the PFK is fructose 2,6-bisphosphate ([Yalcin et al, 2009](#_ENREF_13)), which is synthesized from F6P by phosphofructokinase-2. For simplicity it is assumed that F6P and fructose 2,6-bisphosphate are linearly positively correlated in their dynamics through a fast and reversible phosphofructokinase-2 activity, which was implemented as a Hill kinetic (4 subunits) with a sole activation by F6P. During development of the model, other less efficient regulators such as ATP, ADP, AMP or citrate (data see [Rehberg et al (2013)](#_ENREF_9)) were tested, and finally not considered as the model fit was not improved.

**Transaldolase and transketolase (TATK, EC number: 2.2.1.2 and 2.2.1.1):** both enzymes are responsible for the reversible conversion of metabolites of the PPP, which involve F6P and glycerine-aldehyde phosphate. Because of the unknown flux distribution and unknown concentrations of other PPP metabolites we decided to use individual reversible first order rate laws for F6P and glycerine-aldehyde phosphate formation and degradation. As the latter is not explicitly incorporated in the model, we assumed a strong correlation to 3PG (see also “Aldolase”) and, hence, coupled the activity of the transaldolase and transketolase to 3PG. The chosen reversible mass action kinetics covers the consumption of both intermediates as well as their production by the PPP. We additionally assumed the equilibrium between consumption and production to depend on the corresponding level of F6P and 3PG as well as a threshold defined by $k_{TATKF6P}^{eq}$ and $k_{TATK3PG}^{eq}$, respectively.

**Aldolase (ALD, lumped reaction):** as dihydroxy acetone phosphate, glycerine-aldehyde phosphate and 1,3-bisphosphoglycerate were not quantified for MDCK cells, we assumed that the enzymes, which are involved in the generation of 3PG, are highly correlated with the ALD activity and that the pool sizes are rather low. Again, a Michaelis-Menten kinetic was used to describe the activity of the ALD. As before, a larger model that integrated dihydroxy acetone phosphate, glycerine-aldehyde phosphate and 1,3-bisphosphoglycerate required at least 7 additional parameter while showing only a minor improvement in fitting the data.

**Enolase (ENO, lumped reaction):** we assumed that isomerization of 3PG to 2-phospho glycerate is a very fast and reversible step, which enables describing the generation of PEP from 3PG via 2-phospho glycerate with a reversible first order rate law.

**Pyruvate kinase (PK, EC number: 2.7.1.40):** the pyruvate kinase is represented by a Michaelis-Menten kinetic with activation by F16BP. Consideration of the cofactors ATP and ADP yielded a model fit, similar to the one shown in this work and was, hence, not considered in the model.

# References

Boiteux A, Hess B (1981) Design of glycolysis. *Philos Trans R Soc Lond B Biol Sci* **293:** 5-22

Fitzpatrick L, Jenkins HA, Butler M (1993) Glucose and glutamine metabolism of a murine B-lymphocyte hybridoma grown in batch culture. *Appl Biochem Biotechnol* **43:** 93-116

Fromm HJ, Zewe V (1962) Kinetic studies of yeast hexokinase. *J Biol Chem* **237:** 3027-3032

Janke R, Genzel Y, Händel N, Wahl A, Reichl U (2011) Metabolic adaptation of MDCK cells to different growth conditions: effects on catalytic activities of central metabolic enzymes. *Biotechnol Bioeng* **108:** 2691-2704

Link H, Kochanowski K, Sauer U (2013) Systematic identification of allosteric protein-metabolite interactions that control enzyme activity in vivo. *Nat Biotechnol* **31:** 357-361

Luni C, Marth JD, Doyle FJ (2012) Computational Modeling of Glucose Transport in Pancreatic β-Cells Identifies Metabolic Thresholds and Therapeutic Targets in Diabetes. *PLoS One* **7:** e53130

Neermann J, Wagner R (1996) Comparative analysis of glucose and glutamine metabolism in transformed mammalian cell lines, insect and primary liver cells. *Journal of Cellular Physiology* **166:** 152-169

Otto M, Heinrich R, Jacobasch G, Rapoport S (1977) A mathematical model for the influence of anionic effectors on the phosphofructokinase from rat erythrocytes. *Eur J Biochem* **74:** 413-420

Rehberg M, Rath A, Ritter JB, Genzel Y, Reichl U (2013) Changes in intracellular metabolite pools during growth of adherent MDCK cells in two diffferent media. *Appl Mircobiol Biotechnol*

Renner ED, Plagemann PG, Bernlohr RW (1972) Permeation of glucose by simple and facilitated diffusion by Novikoff rat hepatoma cells in suspension culture and its relationship to glucose metabolism. *J Biol Chem* **247:** 5765-5776

Rivenzon-Segal D, Rushkin E, Polak-Charcon S, Degani H (2000) *Am J Physiol Endocrinol Metab* **279:** E508--E519

Schmid G, Blanch HW (1992) Extra- and intracellular metabolite concentrations for murine hybridoma cells. *Appl Microbiol Biotechnol* **36:** 621-625

Yalcin A, Telang S, Clem B, Chesney J (2009) Regulation of glucose metabolism by 6-phosphofructo-2-kinase/fructose-2,6-bisphosphatases in cancer. *Exp Mol Pathol* **86:** 174-179
