# Supplementary material for: Glycolysis Is Governed by Growth Regime and Simple Enzyme Regulation in Adherent MDCK Cells
Source: PLoS Comput Biol. 2014 Oct 16;10(10):e1003885. doi: 10.1371/journal.pcbi.1003885 (PMC4211564; doi:10.1371/journal.pcbi.1003885)
Supplement: Supporting Information S4 — Predicting the glycolytic activity during cell growth in DMEM medium. (DOCX) [file pcbi.1003885.s013.docx]

# Supporting information 4: predicting the glycolytic activity during cell growth in DMEM medium

## 1 Adjusting the cell growth model

A model for cell growth described by [Rehberg et al (2013)](#_ENREF_2) was comprehensively validated with data from three independent cultivations in GMEM-Z medium. In that work, the well-surface was identified as the main limiting factor of growth. For MDCK cell growth in DMEM medium the original model was modified to account for cell growth under depletion of glucose. Furthermore, it was taken into account that the total cell volume reached by the cells was lower at the end of cultivation ($V_{*}^{C}=5.20\text{ μL}$). Accordingly, the model used in this study was modified as follows. Firstly, the specific growth rate of the cells was considered to depend on the extracellular glutamine concentration (*GLN^x^*) instead of the glucose concentration:

$$\mu=\frac{\mu_{\max}[\mathrm{GLN}^{x}]}{k_{GLN^{x}}^{m}+[\mathrm{GLN}^{x}]}$$

with $\mu_{\max}$ as maximum specific growth rate and $k_{GLN^{^{x}}}^{m}=0.04$mmol L^-1^ as the Monod constant. Secondly, under low glucose concentrations, the uptake typically depends on the affinity of GLUT, which yields a smooth depletion dynamics. To account for such an influence, the uptake rates for glucose were extended by a Michaelis-Menten kinetic for glucose ([Pirt, 1975](#_ENREF_1)):

$$r_{X/GLC^{x}}=\mu(X_{1}f+\sum_{i=2}^{Nc} X_{i})Y_{X/GLC^{x}}\frac{[\mathrm{GLC}^{x}]}{\mathrm{k}_{GLC^{x}}^{m}+[\mathrm{GLC}^{x}]}$$

$$r_{m/GLC^{x}}=m_{GLC^{x}}V^{C}\Theta([GLC^{x}])\frac{[\mathrm{GLC}^{x}]}{k_{GLC^{x}}^{m}+[\mathrm{GLC}^{x}]}$$

with$r_{X/GLC^{x}}$ as medium volume-specific uptake rate for glucose, *X_i_* as the cell number in class *i* of the segregated growth model (see [Rehberg et al (2013)](#_ENREF_2)), *Nc* as the total number of classes, *f* as growth inhibition factor, $Y_{X/GLC^{x}}$ as cell growth-specific yield coefficient of glucose, $k_{GLC^{x}}^{m}$= 0.24 mmol L^-1^ as affinity constant for glucose, $r_{m/GLC^{x}}$ as medium volume-specific uptake rate of glucose for maintenance, $m_{GLC^{x}}$ as cell volume-specific glucose uptake rate for maintenance, $\Theta([GLC^{x}])$ as step function being 1 for $[GLC^{x}]>0$ and zero otherwise. The values for the parameters $V_{*}^{C}$, $k_{GLN^{x}}^{m}$, $k_{GLC^{x}}^{m}$ as well as the initial conditions (Table 2) were derived by fitting of the model to the data. These minor changes allow capturing the growth dynamics of adherent MDCK cells in numbers, mean cell diameter, and glucose consumption (Fig. S1).

Similarly to the growth of MDCK cells in GMEM-Z, the growth phases were identified based on the relative number of growing cells $(\frac{X_{1}f+X_{2}+X_{3}+X_{4}+X_{5}}{X_{tot}})$ with: growth phase 0 – 40 h (0 – 5 % growth inhibition), intermediate growth phase 40 – 75 h (5 – 95 % growth inhibition), and stationary growth phase 75 – 200 h (95 – 100 % growth inhibition). A total cell number of $2.8\times10^{6}$ is reached towards the end of cultivation, which is lower compared to the GMEM-Z cultures (Fig. S1A). The mean cell diameter starts with 15 µm and reaches a maximum of 19 µm at 24 h of cultivation, which is within the range described for MDCK cells for cultivations in GMEM-Z medium (Fig. S1B). The level of GLC^x^ starts at low levels (2.3 mmol L^-1^) and decreases fast due to an exponentially increasing demand by the cells (Fig. S1C). Despite the depletion of GLC^x^ at about 40 h of cultivation, the total cell volume still increases until 58 h of cultivation (figure not shown), which is likely due to the use of stored internal precursors. Therefore, reduction in the final (total) cell volume $V_{*}^{C}$ may either be explained with the differences between media or be attributed to a delayed effect of the glucose limitation. Note that none of the other substrates and by-products reached growth limiting levels.

## 2 Prediction of R5P and UGLC dynamics

Similarly to F6P and F16BP, the pools of R5P and UGLC showed a higher peak than predicted by the model while the peak-width was correctly predicted (Fig. S2). UGLC showed a second increase with a maximum at 96 h of cultivation, which cannot be explained, neither from the biological nor from the model’s perspective.

# References

Pirt SJ (1975) Principles of microbe and cell cultivation: Wiley, New York.

Rehberg M, Ritter JB, Genzel Y, Flockerzi D, Reichl U (2013) The relation between growth phases, cell volume changes and metabolism of adherent cells during cultivation. J Biotechnol
