# Supplementary material for: Glycolysis Is Governed by Growth Regime and Simple Enzyme Regulation in Adherent MDCK Cells
Source: PLoS Comput Biol. 2014 Oct 16;10(10):e1003885. doi: 10.1371/journal.pcbi.1003885 (PMC4211564; doi:10.1371/journal.pcbi.1003885)
Supplement: Supporting Information S5 — Flow of information and initial conditions for parameter fitting. (DOCX) [file pcbi.1003885.s014.docx]

# Supporting information 5: flow of information and initial conditions for parameter fitting

All simulations rely on the structured model for glycolysis introduced in this study (with parameters to be determined) coupled to the segregated cell growth model with parameters fixed as described by [Rehberg et al (2013)](#_ENREF_1). The experiments conducted are influenced by the status of cells at time of cell harvest or at time of initiation of perturbation experiments. With focus on the determination of parameters of enzyme kinetics and to reduce the overall number of parameters during model fitting, the initial conditions for intracellular metabolite concentrations (metabolic status) were determined once for one cultivation at steady-state (Cult1, t=200) and used for simulation of all three cultivations Cult1–3 and the Pred. cultivation. This assumes that cells achieve a reproducible metabolic status in the stationary growth phase of the precultures, and that batch-to-batch variations during this growth phase are small and have a negligible impact on the overall metabolite pool dynamics of the Cult1–3 simulations. Both assumptions are supported by the fact that the actual data of the three cultivations indeed achieved similar final levels. The simulation of Cult1 was in turn used to determine the initial conditions for the intracellular metabolite concentrations for the Lim1-3 simulations based on individual time points t*. The Lim3 simulation was used to determine the initial conditions for the intracellular metabolite concentrations for the Pulse simulation. The scheme Fig. S3 illustrates how the different experiments were taken into account, and how initial conditions were handled for simulation of cultivation, prediction, and perturbation experiments.

# References

Rehberg M, Ritter JB, Genzel Y, Flockerzi D, Reichl U (2013) The relation between growth phases, cell volume changes and metabolism of adherent cells during cultivation. J Biotechnol
