# Supplementary material for: Glycolysis Is Governed by Growth Regime and Simple Enzyme Regulation in Adherent MDCK Cells
Source: PLoS Comput Biol. 2014 Oct 16;10(10):e1003885. doi: 10.1371/journal.pcbi.1003885 (PMC4211564; doi:10.1371/journal.pcbi.1003885)
Supplement: Supporting Information S6 — Nomenclature for parameter of the segregated cell growth model. (DOCX) [file pcbi.1003885.s015.docx]

# Supporting information 6: nomenclature segregated cell growth model

| **Additional parameters of growth model** | |  |
| --- | --- | --- |
| *f* | growth inhibition factor | - |
| $F_{evap}$ | water evaporation rate | L h^-1^ |
| $m_{GLC^{x}}$ | cell volume-specific glucose uptake rate for maintenance | mmol L^-1^ μL^-1^ min^-1^ |
| *Nc* | number of classes | - |
| $r_{m/GLC^{x}}$ | medium volume-specific uptake rate of glucose for maintenance | mmol L^-1^ min^-1^ |
| $r_{x/GLC^{x}}$ | medium volume-specific uptake rate of glucose for growth | mmol L^-1^ min^-1^ |
| $V_{*}^{C}$ | approximate cell volume for larger times | μL |
| $V^{C}$ | (total) cell volume | µL |
| $X_{i}$ | number of cells in class *i* | cells |
| $Y_{X/GLC^{x}}$ | cell growth-specific yield coefficient of extracell. glucose | mmol^-1^ L^-1^ cells^-1^ |
| $\mu$ | specific growth rate | min^-1^ |
| $\mu_{\max}$ | maximum specific growth rate | min^-1^ |
